# Molecular mechanism of a triazole-containing inhibitor of *Mycobacterium tuberculosis* DNA gyrase

**DOI:** 10.1016/j.isci.2024.110967

**Published:** 2024-09-16

**Authors:** Antoine Gedeon, Emilie Yab, Aurelia Dinut, Elodie Sadowski, Estelle Capton, Aurore Dreneau, Julienne Petit, Bruna Gioia, Catherine Piveteau, Kamel Djaout, Estelle Lecat, Anne Marie Wehenkel, Francesca Gubellini, Ariel Mechaly, Pedro M. Alzari, Benoît Deprez, Alain Baulard, Alexandra Aubry, Nicolas Willand, Stéphanie Petrella

**Affiliations:** 1 Institut Pasteur, Université Paris Cité, CNRS UMR 3528, Unité de Microbiologie Structurale, 75015 Paris, France; 2 Univ. Lille, Inserm, Institut Pasteur de Lille, U1177 - Drugs and Molecules for living Systems, 59000 Lille, France; 3 Cimi-Paris, INSERM U1135, Sorbonne Université, AP-HP. Sorbonne Université, Laboratoire de Bactériologie-Hygiène, CNR des Mycobactéries et de la Résistance des Mycobactéries aux Antituberculeux, 75005 Paris, France; 4 Univ. Lille, CNRS, Inserm, CHU Lille, Institut Pasteur de Lille, U1019 - UMR 8204 - CIIL - Center for Infection and Immunity of Lille, 59000 Lille, France; 5 Institut Pasteur, Université Paris Cité, CNRS UMR 3528, Bacterial Cell Cycle Mechanisms Unit, 75015 Paris, France; 6 Institut Pasteur, Plate-Forme de Cristallographie, CNRS UMR 3528, 75015 Paris, France

**Keywords:** Multidrug resistant organisms, Drugs, Molecular Structure, Microbiology

## Abstract

Antimicrobial resistance remains a persistent and pressing public health concern. Here, we describe the synthesis of original triazole-containing inhibitors targeting the DNA gyrase, a well-validated drug target for developing new antibiotics. Our compounds demonstrate potent antibacterial activity against various pathogenic bacteria, with notable potency against *Mycobacterium tuberculosis* (*Mtb*). Moreover, one hit, compound **10a**, named BDM71403, was shown to be more potent in *Mtb* than the NBTI of reference, gepotidacin. Mechanistic enzymology assays reveal a competitive interaction of BDM71403 with fluoroquinolones within the *Mtb* gyrase cleavage core. High-resolution cryo-electron microscopy structural analysis provides detailed insights into the ternary complex formed by the *Mtb* gyrase, double-stranded DNA, and either BDM71403 or gepotidacin, providing a rational framework to understand the superior *in vitro* efficacy on *Mtb*. This study highlights the potential of triazole-based scaffolds as promising gyrase inhibitors, offering new avenues for drug development in the fight against antimicrobial resistance.

## Highlights

• BDM71403, a triazole-based NBTI, is potent against resistant ESKAPE and *M. tuberculosis*
• BDM71403 is more potent than gepotidacin on *M. tuberculosis* gyrase
• BDM71403 blocks the DNA-gyrase complex generating majorly single strand breaks in DNA
• BDM71403 binds to the same site as gepotidacin with additional interactions

## Graphical abstract

**Figure:**
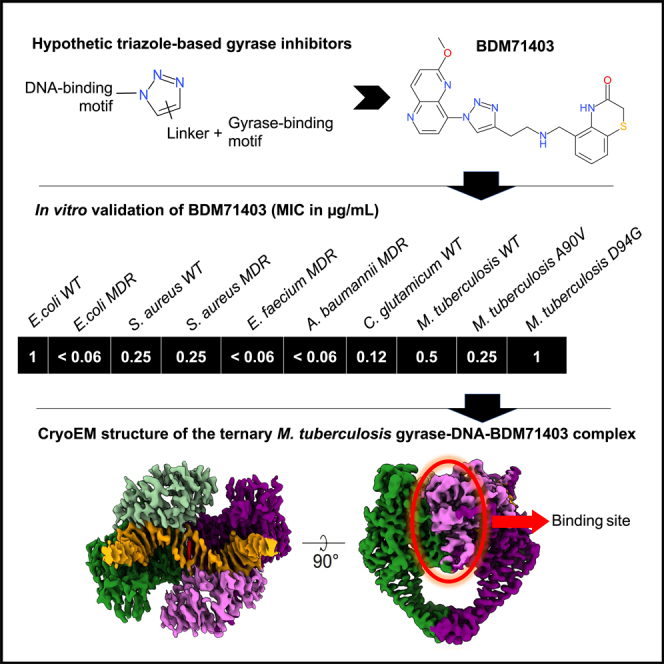

## Introduction

**Figure 1:**
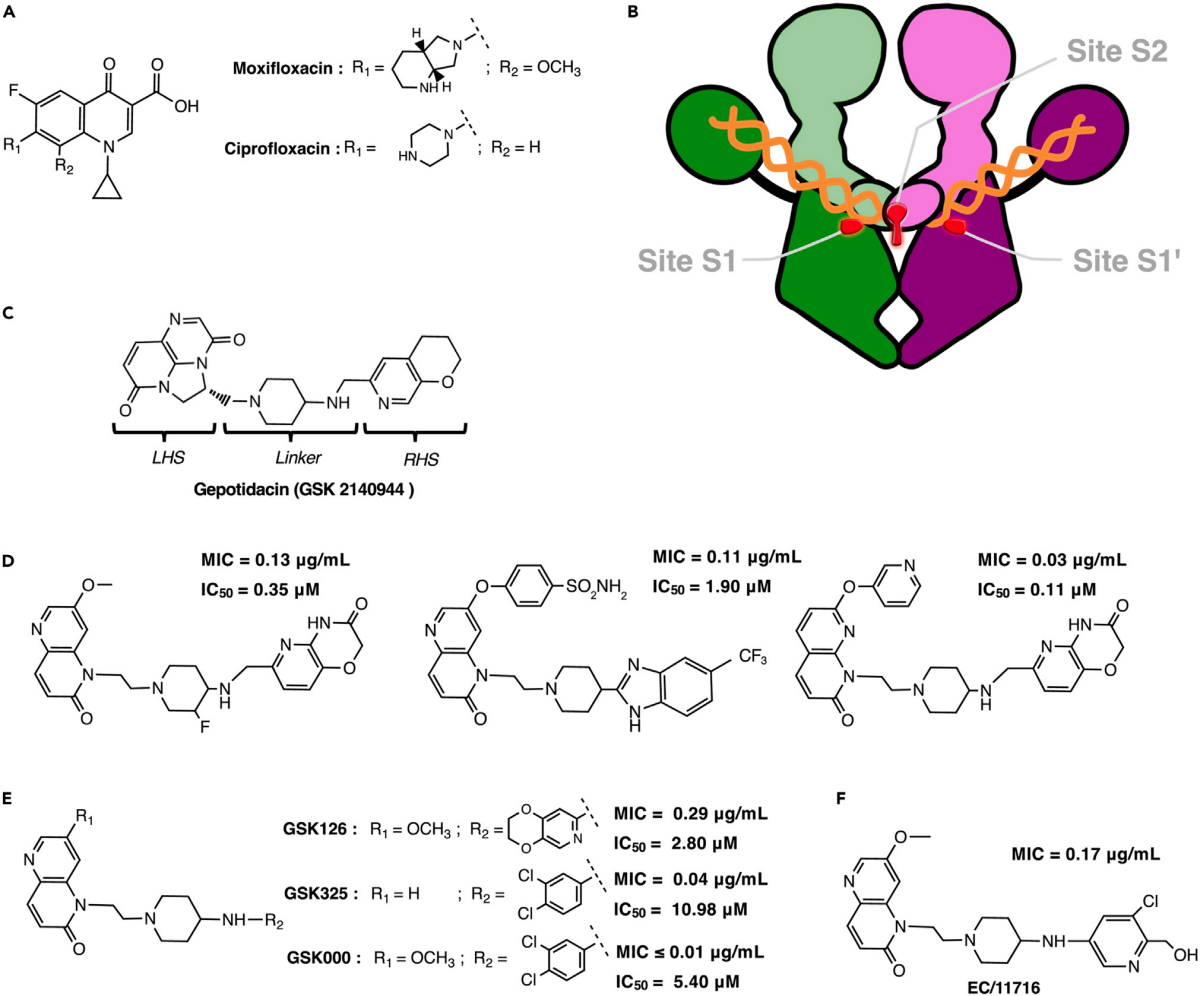
Bacterial type II topoisomerase inhibitors (A) Chemical structures of moxifloxacin and ciprofloxacin, two members of the FQs family. (B) Binding pockets of FQs (sites S1 and S1′) and NBTIs (site S2) within the gyrase-DNA complex. (C) Gepotidacin, also known as GSK 2140944, and representative of the NBTIs family. (D–F) Chemical structures of NBTIs highly potent against *Mtb* identified by AstraZeneca India (D) or (co-)identified by GlaxoSmithKline (E and F).

Despite global control efforts, the latest World Health Organization global tuberculosis (TB) report outlined, like each year, the high incidence rates of multidrug resistant (MDR) *Mycobacterium tuberculosis* (*Mtb*) strains, which are resistant to both isoniazid and rifampicin, the two major anti-TB drugs.^1^ Still amongst the top ten causes of death worldwide, TB is the leading cause of death in patients with HIV, and is responsible for more than one-third of global antimicrobial resistance-related deaths. The main challenges to eradicate TB include discovering and validating new TB targets and identifying potent chemical inhibitors with new mechanisms of action, efficient against multi and extensively drug-resistant TB. Topoisomerases play crucial roles in a number of nucleic acid processes, and are important targets for the development of antimicrobial drugs. Thus, in the context of TB treatment, current second-line regimens include the use of fluoroquinolones (FQs) (i.e., moxifloxacin, the most active FQ against TB, Figure 1A),^2^ a family of synthetic inhibitors specific to type II bacterial topoisomerases (DNA gyrase and Topo IV). Structurally different from human topoisomerases, these enzymes are only found in bacteria and specific bacterial inhibitors (not affecting human topoisomerases) can therefore be identified.^3^^,^^4^

Unlike most other bacteria, the mycobacterium genus lacks the Topo IV enzyme and relies solely on the DNA gyrase for type II topoisomerase activity, thus this enzyme is the only known target for FQs.^5^ The DNA gyrase (5.6.2.2) is a heterotetramer consisting of two GyrA and two GyrB subunits and the only topoisomerase capable of negative DNA supercoiling and compaction in bacteria.^3^ This enzyme can also catalyze relaxation and (de)catenation in a less efficient way by generating 5′-breaks in double stranded DNA.^5^ FQs act as gyrase poisons by binding to two distinct enzyme sites, S1 and S1’ (Figure 1B), and blocking the DNA-gyrase complex.^6^ Unfortunately, the continuous emergence of resistant strains to this family of drugs compromises future use.^7^^,^^8^^,^^9^

More recently, a family of non-quinolone molecules was discovered that is efficient *in vitro* on bacterial DNA gyrase mutants insensitive to FQs. Among these novel bacterial topoisomerase II inhibitors (NBTIs),^10^^,^^11^^,^^12^^,^^13^^,^^14^ the best characterized compound is gepotidacin (or GSK2140944, Figure 1C). Gepotidacin displays high activity against several bacteria, such as *Staphylococcus aureus* and *Escherichia coli*,^15^ and has completed phase II clinical trials for the treatment of acute bacterial skin and skin structure infection.^16^^,^^17^ Currently, gepotidacin is in phase III clinical trials (EAGLE-II/III completed with positive data, a US FDA submission is planned for 2025) set for the treatment of uncomplicated urogenital infection caused by *Neisseria gonorrhea*.^18^^,^^19^^,^^20^

Unlike FQs, gepotidacin binds to a single central site S2 in the gyrase heterotetramer (Figure 1B) and blocks the DNA-gyrase complex in a pre-cleavage state.^21^ This molecule contains a triazaacenapthylene left-hand side (LHS) moiety that binds DNA connected through a nitrogen-containing linker to a pyranopyridine right-hand side (RHS) moiety that binds to protein residues from the GyrA subunit. While the central linker is important for the orientation of the molecule within its binding site, several structural modifications were additionally introduced on the LHS and RHS moieties of gepotidacin to increase its potency against Gram-negative bacteria and to improve metabolic stability.^22^ Further efforts to improve the permeability of these compounds through the complex mycobacterial membrane led to the report of several potent anti-TB NBTIs. AstraZeneca India reported early-on two *N*-linked 1,5-naphthyridin-2-one and one *N*-linked 1H-1,8-naphthyridin-2-one based NBTI gyrase inhibitors with minimum inhibitory concentrations (MIC) and half-maximal supercoiling inhibition concentrations (IC_50_) on *Mtb* gyrase in sub-micromolar ranges (Figure 1D).^23^^,^^24^^,^^25^ A subclass of NBTIs, named novel *Mtb* DNA gyrase inhibitors (MGI), were also developed in parallel by GlaxoSmithKline. Notably, molecules containing a 3,4-dichlorophenyl ring RHS were shown to be highly potent against the tubercle bacillus (*i.e*., MIC lower than 0.01 μg/mL in the case of GSK000) (Figure 1E).^26^^,^^27^ More recently, antibacterial activity of EC/11716, a novel NBTI, on several mycobacterial species, including *Mtb* and *Mycobacterium abscessus*, was reported (Figure 1F).^28^

Interestingly, gepotidacin is different from other NBTIs by the presence of an imidazolidine ring in the left-hand side and the presence of a linker limiting the molecule’s conformational degree of freedom. With this in mind, we chose to investigate the introduction of a triazole ring between the left-hand side and the basic linker via a copper-catalyzed click-chemistry reaction. We then conducted bacterial inhibition assays and *in vitro* DNA gyrase activity assays to characterize the antibacterial activity of these molecules on several bacteria, notably *Mtb*. We further characterized the inhibitory mechanism of action of one of our potent molecules, called BDM71403, against purified *Mtb* gyrase and solved the high resolution cryo-electron microscopy (cryoEM) structures of *Mtb* gyrase bound to DNA and either BDM71403 or gepotidacin, thus deciphering the atomic details of their interactions within the *Mtb* DNA-gyrase complex.

## Results

### Design and synthesis of candidate triazole-based compounds

**Figure 2:**
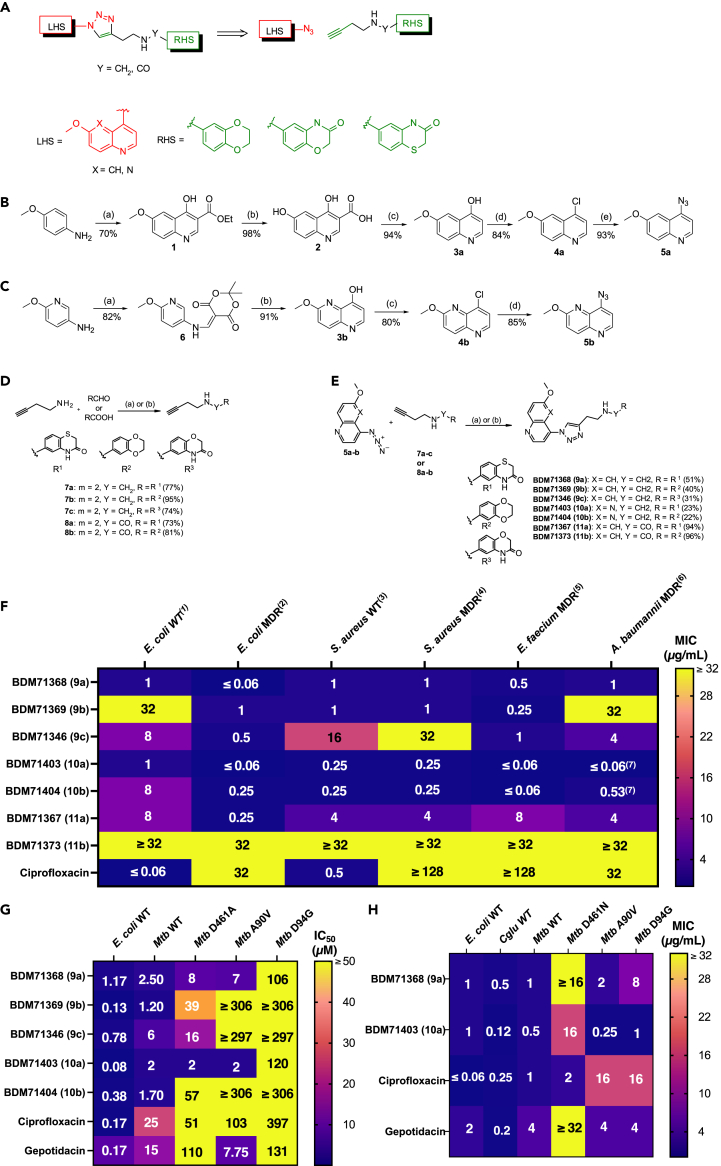
Structure, synthesis and evaluation of *in vitro* efficacy of DNA gyrase 1,2,3-triazoles compounds (A) General scheme of copper-catalyzed azide-alkyne cycloaddition click-reactions. (B) 4-azido-6-methoxy-quinoline synthesis scheme. (***a***) i) Diethylethoxymethylenemalonate, toluene, reflux, 1 h; ii) Eaton’s reagent, 90°C, overnight. (***b***) NaOH, rt, 48 h. (***c***) Diphenylether, 250°C, 30 min (***d***) POCl_3_, 110°C, 1 h. (***e***) NaN_3_, EtOH/H_2_O, 90°C, 24 h. (C) 8-azido-2-methoxy-[1,5]naphthyridine synthesis scheme. (*a*) Meldrum’s acid, triethylorthoformate, ethanol, reflux, 4 h. (***b***) Diphenylether, 250°C, 10 min (***c***) POCl_3_, 110°C, 1 h. (***d***) NaN_3_, EtOH/H_2_O, 90°C, 6 days. (D) Synthesis and structure of alkynes. (*a*) Y = CH_2_; i) RCHO, MeOH/CHCl_3_, MS 4 Å, reflux; ii) NaBH_4_, 0°C then rt overnight. (***b***) Y = CO; RCOOH, EtOAc, T_3_P 50% in EtOAc, 50°C, 5 days. (E) Synthesis and structure of 1,2,3-triazoles. (***a***) Cu(OAc)_2_.H_2_O (20%), sodium ascorbate (40%), *t*-BuOH/H_2_O, 50°C, overnight. (***b***) H_2_O, 110°C, overnight. (F) MIC values (μg/mL) of compounds on wild-type and multiple-resistant ESKAPE strains *E. coli* 8141 wild-type (1), *E. coli* 10385 NDM-1 (2), *S. aureus* 8237 wild-type (3), *S. aureus* 8148 methicillin and FQ resistant (4), *E. faecium* 09001 multi resistant (5), *A. baumannii* 09011 multi resistant (6) and *A. baumannii* 10275 VIM-4 (7). (G) IC_50_ values (in μM) for the inhibition by selected triazoles compounds on DNA supercoiling for wild-type *E. coli* DNA gyrase, and wild-type or FQ-insensitive GyrA mutants of *Mtb* gyrase. (H) MIC values (in μg/mL) of compounds on wild-type *E. coli* DNA gyrase, and wild-type or FQ-mutants of *Mtb* gyrase. MIC values of gepotidacin on wild-type *E. coli* is taken from Biedenbach et al., 2016.^15^ All values represent the mean of three independent measurements (*n* = 3).

Our retrosynthetic approach for the design of title compounds was to connect known azide functionalized LHS (6-methoxy-quinoline and 2-methoxy-[1,5]naphthyridine) with an RHS and a linker containing a terminal alkyne via a copper-catalyzed click-chemistry reaction (Figure 2A). The synthesized compounds can be classified into two series (amine and amide derivatives) according to the nature of the linker.

4-azido-6-methoxy-quinoline **5a** was prepared from *p*-anisidine in 5 steps as shown in Figure 2B. Treatment of *p*-anisidine with diethylethoxymethylenemalonate, followed by subsequent cyclization in the presence of Eaton’s reagent afforded the 6-methoxy-4-hydroxyquinoline-3-ethyl ester 1. Hydrolysis of ester 1 provided the corresponding quinoline carboxylic acid 2, which was decarboxylated in diphenylether to give 6-methoxy-4-hydroxyquinoline **3a**. Subsequent chlorination using phosphorus oxychloride, followed by reaction of chloro derivative **4a** with sodium azide in aqueous ethanol afforded the desired azide **5a** in 50% overall yield.

Synthesis of 8-azido-2-methoxy-[1,5]naphthyridine **5b** is described in Figure 2C. Reaction of 5-amino-2-methoxy-pyridine with Meldrum’s acid and triethyl orthoformate afforded the enamine intermediate 6. The cyclisation was accomplished in boiling diphenylether to give the corresponding hydroxynaphthyridine **3b**, which was converted to azido-derivative following the same procedure as previously. **5b** was thus obtained in 51% overall yield.

Alkynes structures and their synthesis are presented in Figure 2D. Condensation of these amines with the appropriate aldehydes in the presence of molecular sieves, followed by the addition of sodium borohydride as reducing agent allowed us to obtain the alkynes **7a**-**c** in very good yields. Alkynes **8a** and **8b** bearing an amide function were prepared in 73 and 81% yields, respectively, by coupling reaction between the corresponding carboxylic acids and but-3-yn-1-amine, using T3P as activating agent.

The 1,3-dipolar cycloadditions between the two azides and corresponding alkynes were carried out in a mixture of *t*-BuOH/H_2_O at 50°C in the presence of cupric acetate monohydrate and sodium ascorbate as source of copper (I) (Figure 2E). Five compounds (Table 1) have been prepared using this synthetic scheme. These reactions led to 1,4-disubstituted 1,2,3-triazoles in yields ranging from 31 to 96%. It is noteworthy that compounds **10a**-**b** were prepared by thermal cycloaddition, the reactions of azide **5b** with alkynes **7a**-**b** being performed in water at 110°C and leading exclusively to 1,4-regioisomers which were isolated in 23% yield after purification.

**Table 1:** Structure and physicochemical properties of 1,2,3-triazoles compounds

Compound	Chemical structure and chemical formula	Molecular weight (g/mol)	Solubility (μg/mL)	logD
BDM71368 (**9a**)		446.52	84.70	1.81
BDM71369 (**9b**)		417.46	>83.50	1.21
BDM71346 (**9c**)		430.46	76.90	1.32
BDM71403 (**10a**)		447.51	44.00	1.92
BDM71404 (**10b**)		418.45	66.90	1.53
BDM71367 (**11a**)		460.51	<0.17	>2.65
BDM71373 (**11b**)		431.44	10.20	2.35

Solubility and logD values were measured as described in STAR Methods.

As expected, analogues from the amine series (**9a-9c** and **10a-b**) showed higher solubilities (>44 μg/mL) than amides (<11μg/mL) and lower logD.

### Evaluation of potency on bacterial growth of ESKAPE strains

To initially evaluate antibacterial activity of the compounds, we measured MICs on several wild-type and MDR ESKAPE^29^ strains (*Enterococcus faecium*, *Staphylococcus aureus*, *Klebsiella pneumoniae*, *Acinetobacter baumannii*, *Pseudomonas aeruginosa* and *Enterobacteriales* spp., *i.e*., *E. coli*). Several of our synthesized compounds exhibited *in vitro* antibacterial activity against wild-type or quinolone resistant strains. Triazoles **9a**, **9b**, and **9c** that share the same 6-methoxyquinoline LHS connected to different RHS (1,4-benzothiazin-3-one, 2,3-dihydro-1,4-benzodioxine, 1,4-benzoxazin-3-one and 1,3-benzodioxole rings respectively) present moderate to mild antibacterial potency (Figure 2F). Molecules harboring a 1,5-naphthyridine LHS (molecules **10a** and **10b**) showed the broadest antibacterial activity. For instance, with the molecule **10a**, MIC evaluation showed 32-fold and at least 128-fold improvement over FQs in the case of quinolone resistant *E. coli* and *S. aureus* respectively (Table S1). Notably, replacement of the amine linker with an amide function (molecules **11a** and **11b**), led to less potent analogues.

### Evaluation of anti-mycobacterial potency on *Mtb* DNA gyrase inhibition and bacterial growth of selected compounds

Five molecules of interest (**9a-c** and **10a-b**) were further tested *in vitro* for their potency to inhibit the *Mtb* gyrase. First, we measured IC_50_ values by monitoring the alteration of isomerization of relaxed DNA to supercoiled DNA. Activity assays on wild-type *E. coli* and *Mtb* gyrases revealed a potent inhibition for all five molecules, validating their mode of action as gyrase inhibitors (Figure 2G). Interestingly, we observed that IC_50_ values for compounds **9a** and **10a**, both harboring a 1,4-benzothiazin-3-one RHS unit, are 10- and 12-fold lower than the values measured for ciprofloxacin and are 6- and 7-fold lower than those of gepotidacin on wild-type *Mtb* gyrase (Figure 2G).

To further characterize the inhibitory activity on *Mtb* FQ-resistant GyrA or GyrB mutants, we considered two *Mtb* GyrA and one *Mtb* GyrB variants carrying single missense substitutions at residues lining the binding sites S1 and S1′ of FQs (positions 90 or 94 in *Mtb* GyrA, or 461 in *Mtb* GyrB, all linked to more than 90% of FQ-resistances in *Mtb* clinical isolates^30^). Again, IC_50_ values for compounds **9a** and **10a** reflected ameliorated potency on mutants D461 and A90 in comparison with inhibition by ciprofloxacin and gepotidacin for *Mtb* gyrase (Figure 2G). Nonetheless, our results revealed compound **10a** as the most potent hit to inhibit the activity of wild-type gyrases and mutants carrying substitutions leading to FQ resistance.

To corroborate this observation on antimycobacterial activity, we measured the MICs of these two hits on *Mtb* wild-type and FQ resistant strains harboring genomic substitutions for each of the three residues of interest (Figure 2H). Gepotidacin was also included in the assays, as no comparative data was available in the literature for *Mtb*. Several important new observations were made in these experiments. First, gepotidacin was mildly effective against *Mtb* strains, and indeed molecules **9a** and **10a** were more potent than gepotidacin against wild-type *E. coli* and *Mtb* strains. Second, cross-resistance for the three molecules were observed in FQ-resistant strains with a GyrB D461 substitution (at least an 8-fold increase of MIC when compared to the wild-type *Mtb* strain for gepotidacin). These observations are complementary to the IC_50_ evaluations. Thus, molecule **10a** is the compound with the lowest MIC value for bacteria resistant to FQs (Figure 2H). Finally, the correlation curve between IC_50_ and MICs determined for each drug tested on the *Mtb* wild-type strain confirmed that compound **10a** (BDM71403) has the same characteristics than the more effective FQ like levofloxacin and moxifloxacin. Otherwise, the comparison with gepotidacin shows a lesser decrease in intracellular concentration^31^^,^^32^ (Figure S1).

### Inhibition mechanism of BDM71403 on *Mtb* gyrase and *in vitro* competition evaluation with fluoroquinolones

**Figure 3:**
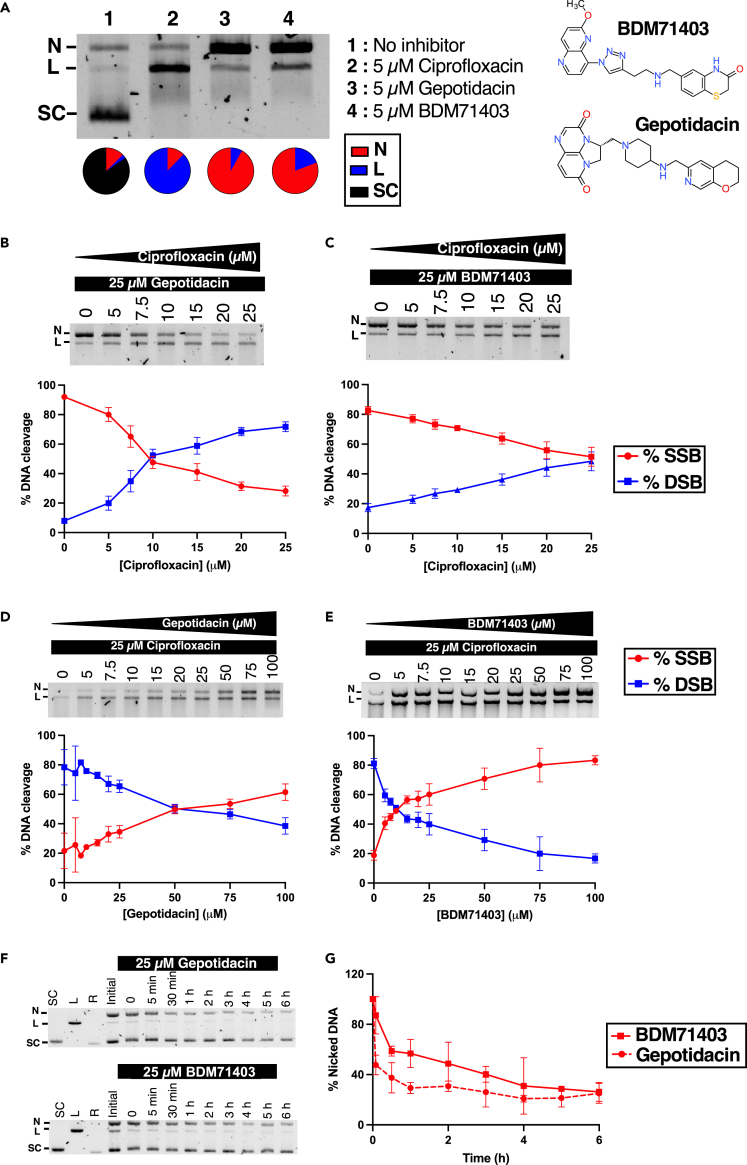
Mechanism of inhibition of BDM71403 on *Mtb* gyrase (A) DNA cleavage assay on pBR322 (5 nM) with *Mtb* gyrase (200 nM) in the absence (lane 1) or the presence of 5 μM ciprofloxacin (lane 2), gepotidacin (lane 3) or BDM71403 (lane 4). Chemical structures of BDM71403 and gepotidacin are depicted in the right side for comparison. (B–E) DNA cleavage competition on *Mtb* DNA gyrase (200 nM) via formation of single-strand breaks (SSB, in red) or double-strand breaks (DSB, in blue) on pBR322 (5 nM) in the presence of variable concentrations of ciprofloxacin and (B) 25 μM gepotidacin or (C) 25 μM BDM71403; or in the presence of 25 μM of ciprofloxacin and variable concentrations of (D) gepotidacin or (E) BDM71403. Bands corresponding to nicked and linear DNA were quantified and reported as percentages for each condition in the corresponding graph. (F and G) Time course of cleavage complex stability of *Mtb* gyrase in the presence of supercoiled DNA and 25 μM gepotidacin or BDM71403. *Mtb* gyrase-DNA stability was evaluated by a cleavage assay after 20-fold dilution of reaction mixture as described in STAR Methods (1 μM of *Mtb* gyrase and 100 nM of pBR322) and shown as a gel (F) and a graph reporting the percentage of nicked DNA as a function of time (G). The “Initial” wells represent the reaction mixtures prior to the 20-fold dilution. All points and error bars represent mean and standard deviations from three independent experiments (*n* = 3). N, nicked DNA; L, linear DNA; SC, supercoiled DNA; R, relaxed DNA.

NBTIs and FQs were previously shown to trap the ternary DNA-gyrase-inhibitor complex via different mechanisms. NBTIs induce majorly single-strand nicked DNA breaks (although minor double-strand breaks have been also shown to be induced by dioxane-linked amide derivatives NBTI compounds in the case of topoisomerase IV^33^^,^^34^^,^^35^); unlike the double-strand DNA breaks induced by FQs. To evaluate the mode of action of BDM71403, we first employed a DNA cleavage assay and discerned the different DNA states mediated by *Mtb* gyrase on supercoiled DNA in the presence of the drug. Gepotidacin and ciprofloxacin were considered representative controls of NBTIs and FQs respectively. Cleavage assays were conducted on a *Mtb* GyrBA fusion protein, since this chimera has the same specific activity as the non-fusion *Mtb* gyrase and can be expressed and purified with a higher yield.^36^ As shown in Figure 3A, the presence of BDM71403 (lane 4) resulted in a major population of nicked DNA, similar to that of gepotidacin (lane 3), and different from ciprofloxacin (lane 2). As a control, no DNA cleavage occurred in the absence of the enzyme (Figure S2). Interestingly, a minor population (10–20%) of linearized DNA was induced by gepotidacin or BDM71403. The level of linearized DNA slightly increased with higher concentrations of the inhibitor, as demonstrated by titration with increasing inhibitor concentrations (Figure S3). These observations validate that the mechanism of inhibition of BDM71403 is similar to that of gepotidacin.

Previous reports on *Mtb* gyrase and *S. aureus* gyrase described non-synergetic inhibition between NBTIs and FQs, despite their ability to bind to separate sites within the DNA-gyrase complex (Figure 1B).^21^^,^^27^ To investigate inhibitory competition between BDM71403 and ciprofloxacin on the *Mtb* gyrase, we employed the same cleavage assay with saturating concentrations of one inhibitor and variable concentrations of the other, using gepotidacin as a control. Assays were conducted in saturating concentrations of the enzyme (200 nM) to favor the complete conversion of supercoiled DNA substrate, and therefore disperse DNA into only linear and/or nicked DNA populations (Figure S4). Band intensities were quantified to evaluate the percentage of linear and nicked DNA per condition. The results showed that increasing concentrations of ciprofloxacin in the presence of saturating concentrations of gepotidacin or BDM71403 disfavor the formation of nicked DNA and induce the formation of linear DNA. Notably, gepotidacin is more easily displaced by ciprofloxacin than BDM71403, as the drop of nicked DNA to 50% is induced by a 2.5-fold lower concentration of BDM71403 in comparison with gepotidacin (10 μM vs. 25 μM, Figures 3B and 3C). The same observations can be noted for the reverse experiment with saturating concentrations of ciprofloxacin where nicked DNA is diminished by 50% at 10 μM BDM71403 or 50 μM gepotidacin (Figures 3D and 3E). Taken together, these observations show that BDM71403 inhibits gyrase activity as an NBTI, exhibits a higher binding affinity than gepotidacin to the *Mtb* gyrase-DNA complex, and cannot occupy the same cleavage complex with FQs.

### Persistence of the *Mtb* gyrase-DNA-BDM71403 complex

To evaluate the BDM71403-induced *Mtb* gyrase-dependent DNA cleavage stability, we employed a DNA cleavage-religation assay^37^^,^^38^^,^^39^ in which reactions were carried out similarly to the cleavage assays in the presence of saturating concentrations of BDM71403 or gepotidacin with higher quantities of substrate and enzyme. Samples from different time points were treated and analyzed as cleavage assays (Figure 3F). Bands corresponding to nicked DNA were quantified to follow the dilution-mediated decay of the cleavage complex (DNA religation) as a function of time (Figure 3G). Results showed that the cleavage complex is more stable in the presence of BDM71403 (half-life of the complex t_1/2_ = 0.75 h) than in the presence of gepotidacin (t_1/2_ = 0.07 h). This 10-fold difference in the complex half-life is significant (*p* = 0.02) and reflects the improved stability of the BDM71403-bound tripartite complex.

### Structural analysis of the *Mtb* gyrase-DNA complexes in the presence of BDM71403 or gepotidacin

**Figure 4:**
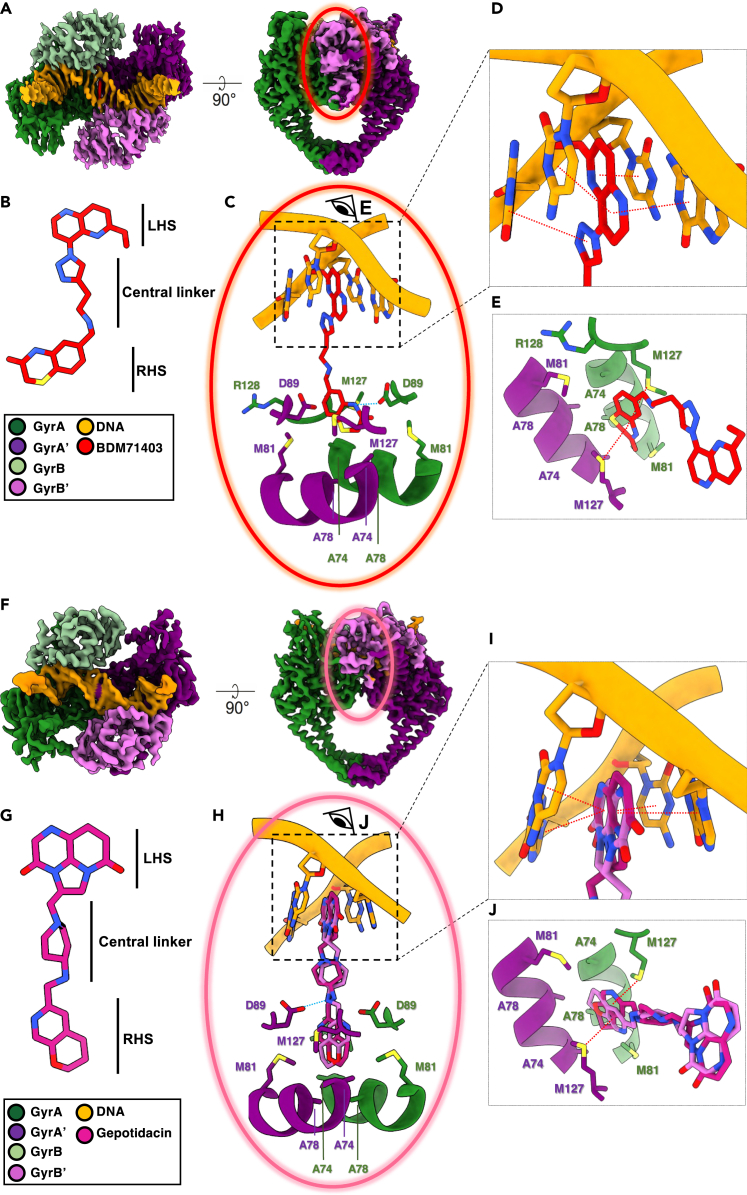
BDM71403 and gepotidacin binding pockets (A, F) CryoEM maps of the cores at high resolution, including BDM71403 and gepotidacin, are colored according to code shown in this panel. (B, G) Structures of BDM71403 and gepotidacin. (C, H) Close-up view of the drugs binding sites. Hydrogen bonds are represented as dotted blue lines, and residues forming the binding pocket are represented as sticks and labeled. The two possible orientations of gepotidacin are depicted in medium violet and orchid stick representation. (D, I) Details of the π-stacking interaction represented as red dotted lines between nucleobases, LHS and triazole substituents. (E, J) Top-view of the drugs binding sites with the sulfur-aromatic-stacking interaction represented as red dotted lines. DNA and non-useful residues composing the binding site were removed for better visibility.

To understand the detailed binding properties of the drugs to the *Mtb* gyrase-DNA complex, we collected two cryoEM datasets of the *Mtb* GyrBA fusion in complex with a 150 bp double-strand DNA (dsDNA) and either BDM71403 or gepotidacin. CryoEM density maps were obtained at an overall resolution of 2.8 Å (BDM71403-bound) and 3.1 Å (gepotidacin-bound) (Figure 4; Figures S5 and S6 and Table S2). All secondary structure elements and residue side chains could be unambiguously assigned. As reported for other NBTIs in gyrase orthologs,^14^^,^^21^^,^^40^ both gepotidacin and BDM71403 bind to the same central site (site S2, Figure 1B), halfway between the two 4bp-separated FQs binding sites (sites S1 and S1′, Figure 1B).^41^ As previously shown for this group of compounds, only one molecule is bound to stabilize the DNA-gyrase complex (Figure 4). Neither cleaved dsDNA nor cleaved ssDNA could be observed in our structures (Figures 4 and S7A). The superposition of the cleavage cores of the BDM71403-and gepotidacin-bound complexes show 1.2 Å RMSD for all C_α_ atoms of the cleavage cores, and an almost perfect alignment of the two DNA fragments. Superposition of these structures with the *Mtb* cleavage core in complex with FQ showed 1.4 Å RMSD for C_α_ atoms, indicating that both drugs block the cleavage core in a closed conformation as already described for *Mtb* gyrase:dsDNA:FQ complexes (Figure S6).^41^

In both inhibitor-bound structures, the LHS substituent sits in a pocket formed of base pairs from the dsDNA on the 2-fold axis of the complex, whereas the RHS part sits in a pocket on the 2-fold axis between residues from the two GyrA subunits (Figures 4C and 4H). It is interesting to note that the LHS binding pocket in the DNA does not exist in the binary complex of double-stranded cleaved DNA of the *Mtb* DNA gyrase, implying that slight reorientations of base pairs must take place to accommodate the inhibitor.^41^ Common interactions shared by gepotidacin and BDM71403 are the π–π stacking of the LHS part with nucleobases, promoting only a subtle spacing difference between nucleotides (Figure S6B), and the van der Waals interactions between the RHS part and the highly hydrophobic pocket formed of residues Ala74, Ala78, Met81 and Met127 from the two GyrA subunits Figures 4C and 4H). A conserved hydrogen bond interaction is observed between Asp89 form GyrA and the amine from the central linker of gepotidacin,^21^ but is not visible in the BDM71403-bound complex where an indirect hydrogen bond via a water molecule might exist.

Despite the overall structural similarity of the complexes, slight differences are observed between the two compounds. We will focus below on the BDM71403-bound structure. In this complex, the 1,4-naphthyridin-2-one LHS of the molecule and the triazole ring are maintained in contact with DNA via π–π stacking interactions of nucleobases (Figure 4D). A hydrogen bond between Asp89 and the nitrogen atom of the RHS fixes the orientation of the molecule inside the binding cavity (Figure 4C), further stabilized via van der Waals interactions of the carbonyl group of the RHS moiety with the carbonyl chain of Arg128 (Figure 4E). We also observed sulfur-aromatic interactions between the aromatic ring in the RHS and the sulfur atoms of Met127 from the GyrA subunit (Figures 4D and S6C). It’s interesting to note that the three sulfur atoms coming from Met127 from GyrA (chains A and B) and from the RHS dihydrothiazine substituent, are in a parallel orientation and separated by a distance of 4.6 Å suggesting also possible sulfur-sulfur interactions^42^ (Figures 4D and S6C). In the gepotidacin-bound structure, the same type of sulfur-aromatic interactions is visible for the RHS part without the presence of the third sulfur atom coming from the RHS substituent (Figure 4I). It is notable that gepotidacin adopts two distinct conformations within the S2 site, also observed in the crystallographic structure of the *S. aureus* gyrase-gepotidacin complex,^21^ whereas in our BDM71403-bound structure only one possible binding on the 2-fold axis of the complex was observed. Possibly related to the relative stabilities of the two inhibitor complexes, the side chain of Arg128 adopts a different orientation. This Arg128, which forms one side of the drug binding cavity, is close to the Mg^2+^ ion essential for the activity of gyrase. Analyzing the cryoEM maps at various contouring levels suggests the absence of this ion in the BDM71403-bound structure, while it is present in the gepotidacin-bound structure, albeit with a slightly different position in the two chains (Figures S7B and S7C).

### Effect of BDM71403 on bacterial cell morphology

To further validate the mode of action of BDM71403, we evaluated its impact on the morphology of bacteria using the non-pathogenic *Corynebacterium glutamicum* (*Cglu*) ATCC1332 strain, as a surrogate for *Mtb. Cglu* has been extensively used as a model to investigate conserved core mechanism of Corynebacterineae cell physiology, such as cell envelope synthesis.^43^^,^^44^^,^^45^ *Cglu* and *Mtb* exhibit a close phylogenetic relationship^46^ and possess comparable cell wall architectures, a crucial factor influencing drug penetrability. As in *Mtb*, the *Cglu* DNA gyrase is also the sole type II topoisomerase present in the genome and is highly similar (80% sequence similarity). Additionally, the MIC values for FQs and BDM71403 are similar between *Mtb* and *Cglu* (Figure 2H).

**Figure 5:**
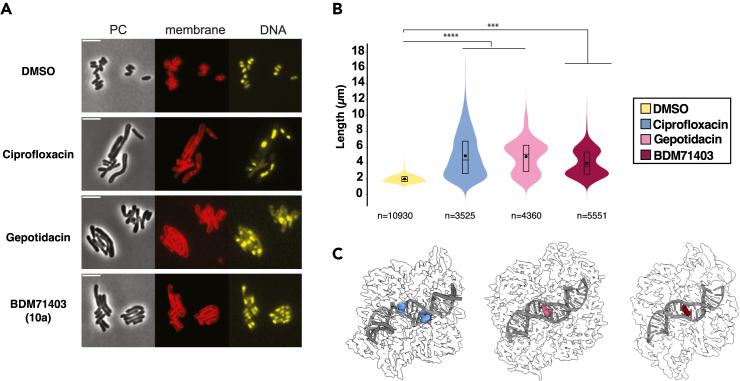
Evaluation of the impact of gyrase inhibitors on the phenotype of wild-type *C. glutamicum* ATCC1332 strain (A) Representative images in phase contrast (PC) with Nile Red (membrane marker, in red) and Hoechst (DNA marker, in yellow) of wild-type *C. glutamicum* in the absence (DMSO, control) or in the presence of each indicated drug. Scale bars correspond to 5 μm. (B) Violin plots showing the distribution of cell length for each tested condition from a. (Cohen’s *d*, from left to right when compared to control: (∗∗∗∗, *d*(ciprofloxacin) = 2.08; ∗∗∗∗, *d*(gepotidacin) = 2.36; ∗∗∗, *d*(BDM71403) = 1.87). The box indicates the 25^th^ to the 75^th^ percentile and the whiskers indicate the 95% confidence interval. Mean and median are indicated with a dot and a line in the box. The number of cells (n) considered for the analysis are indicated below each violin. (C) Binding sites for FQs, gepotidacin and BDM71403 highlighted on the gyrase-DNA complex structures.

To assess the effects of BDM71403 as well as those of the reference molecules for FQs (ciprofloxacin) and NBTIs (gepotidacin) on cell morphology, the molecules were added to an early exponential bacterial growth culture and visualized under the microscope. All three gyrase inhibitors showed strong morphological defects when compared to the control strain (*Cglu* in the presence of DMSO). The cells were elongated and DNA segregation severely affected (Figures 5A and 5B). Interestingly there appears to be a distinctive phenotype between ciprofloxacin and the two drugs, gepotidacin and BDM71403. Ciprofloxacin induces extreme cell elongation with a significant number of cells longer than 8 μm when compared to gepotidacin and BDM71403, both of which display a similar morphological pattern. These morphological readouts correlate well with the distinct binding sites observed for FQs and NBTIs in the 3D structures (Figure 5C), suggesting different underlying mechanisms and confirming that BDM71403 activity on the bacteria is similar to NBTIs.

## Discussion

Treating TB requires a constant introduction of novel compounds to combat emerging resistance. The rise of MDR and extensively drug resistant (XDR) *Mtb* strains resistant to most currently available antibiotics underscores the need for new therapies. Since all drugs are introduced as combinations, each new compound added to an existing regimen could provide a solution for treating emerging resistance. Ideally, such combinations should contain novel non-FQ compounds free of preexisting resistance in the case of DNA gyrase inhibition-based treatments. Fortunately, several non-FQ gyrase inhibitors with novel inhibitory modes have been recently discovered, paving the way for the investigation of new binding pockets. Miller et al. discovered in 2008 quinoline pyrimidine trione-1 (QPT-1, also known as PNU-286607) as an antibiotic against *E. coli* and *S. aureus* via the inhibition of gyrase.^47^ Chan et al. showed in 2015 that QPT-1 is able to bind within the FQ S1 and S1′ sites of *S. aureus* gyrase with a remarkably different binding mode, making it effective toward FQ-resistant variants.^48^ Chan et al. elegantly reported in 2017 yet another family of synthetic thiophene-based molecules that act as gyrase inhibitors and showed effective *in vitro* antibacterial activities against ESKAPE strains.^49^ Thiophenes were shown to bind within the DNA-gyrase complex onto two distinct allosteric sites that are exclusively formed of residues from *S. aureus* GyrA and GyrB subunits, with no interactions with DNA moieties. Other efforts have led to gyrase inhibitors that are efficient against *Mtb*. In 2022, Govender et al. described spiropyrimidinetrione (SPT) inhibitors with potent *in vitro* antitubercular activities (MIC = 0.25 μg/mL; IC_50_ = 2 μM).^50^ Docking experiments predicted binding of SPTs as being identical to QPT-1. In parallel, Imai et al. identified evybactin, a nonribosomal peptide from the animal pathogenic bacteria *Photorhabdus noenieputensis*, as being effective *in vitro* against *Mtb*.^51^ A crystallographic structure of the cleavage core of *Mtb* gyrase in complex with DNA and evybactin showed that this peptide binds with a stoichiometry 1:1:1 onto a pocket that overlaps with one of the thiophene sites.

Aminopiperidine-based NBTI, represented by gepotidacin, are the most explored scaffolds to-date for the development of antibacterial compounds acting as gyrase inhibitors. Despite validation of a broad *in vitro* antibacterial spectrum for gepotidacin against *S. aureus*, *E. coli*, *S. pneumoniae*^52^ and *N. gonorrhea*,^53^ and more recently extended to *Francisella tularensis*,^54^ *Yersinia pestis*,^55^ nosocomial pathogen *Stenotrophomonas maltophilia*,^56^ and several anaerobic infectious bacteria (such as *Fusobacterium* spp., *Actinomyces* spp. and *Clostridium* spp.),^57^ no antitubercular activity for this molecule was reported by *in vitro* screening of large NBTIs libraries on *Mtb*^26^ (but subsequently led to the identification of MGIs, Figure 1D).

In this work, we explored new chemical series harboring a triazolethylamine linker and containing bicyclic LHS and RHS moieties. From seven synthesized molecules, five hits were preselected based on their antibacterial potency against wild-type and multidrug resistant ESKAPE strains. All molecules of interest contained [1,4]-thiazine, -dioxine or -oxazine functions in RHS (molecules **9a**, **9b** and **9c**), or 1,5-naphtyridine in LHS (molecules **10a** and **10b**). Antimycobacterial potency was therefore evaluated by measuring supercoiling inhibition on purified wild-type and FQ-resistant mutants of *Mtb* gyrase (GyrB D461, GyrA A90 and D94), and minimal inhibitory concentrations of bacterial growth on wild-type *Mtb* and corresponding gyrase mutant strains manifesting decreased FQ susceptibility. We observed a correlation between IC_50_ and MIC values showing that the most active compound, even on FQ-resistant strains, is molecule **10a** (Figure S1). These results comforted us with the fact that the main target for this molecule is DNA gyrase; however, we cannot rule out potential secondary mechanisms of action for this molecule.

To characterize the molecular mechanism of action of this family of molecules, the most potent molecule against *Mtb*, compound **10a** (BDM71403), was further studied. By using cleavage assays with *Mtb* gyrase, we have proven that this compound stabilizes single-stranded cleavage complexes (Figure 3A), as described for NBTIs.^21^ Being mechanistically different than FQs (for which the induction of double strand DNA breaks has been reported), we conducted cleavage competition assays between ciprofloxacin and BDM71403 on *Mtb* gyrase. These experiments showed a clear non-synergistic activity of these two molecules on *Mtb* gyrase, as manifested by a loss of the BDM71403-induced DNA nicking (Figures 3B and 3C) or the loss of ciprofloxacin-induced linearization of DNA (Figures 3D and 3E). Remarkably, our observations are in accordance with previous competition reports for gepotidacin and moxifloxacin on *S. aureus* gyrase^21^ and the NBTI GSK000 (Figure 1E) and moxifloxacin on *Mtb* gyrase.^27^ Furthermore, by conducting assessments of complex persistence, we evaluated the stability of the *Mtb* gyrase/DNA/inhibitor complex. Our findings indicate an improved half-life of the complex (0.75 h) when BDM71403 is present, as opposed to the reference NBTI gepotidacin (0.07 h). Moreover, BDM71403 showed higher half-life values than reported on wild-type *Mtb* gyrase for ciprofloxacin (0.4 h) and levofloxacin (0.6 h), but not gatifloxacin (2.5 h) and moxifloxacin (3 h) which are the more potent FQs against *Mtb*.^41^ These results could partly explain why BDM71403 is more efficient on *Mtb* than gepotidacin.

To explore BDM71403 binding on *Mtb* DNA gyrase at an atomic level and try to find more evidences illustrating its better potency against *Mtb*, we conducted cryoEM experiments. BDM71403 inhibitor was incubated in the presence of *Mtb* GyrBA and a 150bp dsDNA. In parallel, we managed to solve the structure of *Mtb* gyrase with the same dsDNA in the presence of gepotidacin. Both cryoEM structures revealed ternary complexes gyrase-dsDNA-inhibitor with a stoichiometry of 1:1:1. In *Mtb* gyrase complexes, the fact that the DNA was intact indicates that the compounds stabilize a pre-cleavage enzyme-DNA complex and thus inhibit strand separation. In the BDM71403-bound complex, the absence of the Mg^2+^ ions usually present in the GyrB domain just near the catalytic tyrosines further support the absence of DNA cleavage in this complex (Figure S7A). These ions are essential for the nucleophilic attack of the ds-DNA. In our gepotidacin-bound complex, these ions are present, even if they are not exactly at the same position in the two chains (Figures S7B and S7C). It is important to note that the cryoEM structures we observe represent an average of various conformational states for the enzyme. Nevertheless, as for other reported NBTIs, we observed in our cleavage activity assays that BDM71403 or gepotidacin stabilize single-strand DNA cleavage which means that we have a certain flexibility of the residues in this catalytic pocket but the drugs blocks the transformation of DNA to the asymmetric conformation, thus preventing cleavage of the second strand.^48^^,^^58^^,^^59^ Analysis and comparison of the binding mode of BDM71403 to those of gepotidacin allowed the identification of several additional interactions important to stabilize the drug in one specific orientation in its binding pocket. The main interactions are an additional hydrogen bond between the nitrogen of the linker and Asp89 of GyrA, the localization of RHS carbonyl group in a polar pocket and sulfur-aromatic interactions (4.6 Å) between the RHS aromatic atom and the Met127 sulfur atom of GyrA. It is accepted that the S-aromatic interaction occurs at a greater distance (5–7 Å) than a salt bridge (<4 Å), while the energies associated with either interaction are comparable.^60^^,^^61^ More recently, extensive surveys of the Protein DataBank have revealed the importance of the methionine-aromatic motif for stabilizing protein structures and for protein–protein interactions.^62^^,^^63^ This corroborates the fact that, the chemistry of this substituent in direct interaction with the protein is crucial. Finally, it is also important to note that the presence of the polar triazole structure in the linker allows the precise adaptation of the RHS for π–π stacking with dsDNA. This highlights a superior stabilization of BDM71403 in comparison to gepotidacin, elucidating the 10-fold longer half-life in the BDM71403-bound complex compared to the gepotidacin-bound complex.

These data also allow a better understanding of how the substitutions implicated in FQs resistance could have an impact on the binding of NBTIs. Indeed, the analysis of the BDM71403-bound structure reveals that the three substitutions implicated in FQ resistance (GyrA A90 and D94, and GyrB D461) are all located at relative long distances from the drug (8–11 Å - Figure S8A), implying an indirect effect of these substitutions on both the stabilization of the drug and the lower sensibility of the substituted gyrases to BDM71403. GyrA substitutions of A90 and D94 into residues with increased steric hindrance and reduced polar proprieties result in alterations of the binding pocket environment. This, in turn, has the potential to influence the network of water molecules around the drug. This hypothesis is further supported by the fact that IC_50_ values of BDM71403 for *E. coli* wild-type gyrase (0.08 μM) is 25-fold lower than that for the *Mtb* gyrase (Figure 2G), suggesting a higher binding affinity in the former case. Interestingly, in *E. coli,* in place of an alanine, a serine is found at position 90 in GyrA (Figure S9). In the context of BDM71403, the presence of this serine further underscores the idea that a polar residue at this position could play a crucial role in the water network. Regarding the influence of the GyrB substitution, it may directly contribute to DNA stabilization, thereby influencing the formation of the pocket within the dsDNA molecule. Additionally, D461 serves as a crucial residue in network of hydrogen-salt bridges implicated in regulating the dimerization of the ATPase domains. Consequently, it could play a significant role in the overall enzyme function by controlling this dimerization and subsequently altering the accessibility of the drug-binding pocket (Figure S8B). Imai et al.’s recent research has also provided further evidence supporting the connection between diminished sensitivity to other drugs and resistance to FQs through distal binding site interactions.^51^

Based on these findings, it can be asserted that the triazole-based BDM71403 belongs to the NBTIs subclass and has a greater potency against *Mtb* gyrase in comparison to NBTIs such as gepotidacin. This also confirmed that gepotidacin has a compromised efficacy on *Mtb* wild-type and FQ-mutated strains, via the existence of cross-resistance between this drug and FQs. Notably, the extent of cross-resistance is markedly reduced for BDM71403. Overall, this study provides valuable insight into the development of novel triazole-based NBTIs scaffolds acting as DNA gyrase inhibitors with very low cross-resistance with FQs.

### Limitations of the study

We report a rational design and characterization of BDM71403 as a potent antimycobacterial drug. Our results prove that BDM71403 acts as a DNA gyrase inhibitor, rendering it a new member of NBTIs with a never-before explored triazole linker. The specificity of this molecule to other potential targets remains however a pending question; a selection of resistant bacteria to this drug could provide more detailed insight into the mode of action of BDM71403 in cellular contexts.

We have shown that this drug induces the generation of single-stranded cleaved DNA by DNA gyrase, whereas the DNA stabilized in the 3D structure obtained by cryo-EM is not cleaved. Further studies will be necessary to explain this difference.

## Resource availability

### Lead contact

Further information and requests for resources and reagents should be directed to and will be fulfilled by the lead contact, Stephanie Petrella (stephanie.petrella@pasteur.fr).

### Materials availability

Materials will be made available from lead contact upon request.

### Data and code availability

• All data reported in this paper will be shared by lead contact on request.
• This article does not report original code.
• Any additional information required to reanalyze the data reported in this paper is available from lead contact upon request.

## Acknowledgments

The authors are grateful to the staff of the Nanoimaging facility for assistance in cryoEM experiments. This work benefited from access to the cryoEM platform of the European Molecular Biology Laboratory (EMBL) in Heidelberg, an Instruct-ERIC center. We thank Christel Neut and Luc Dubreuil for their support in in vitro characterization of the compounds. We thank Claudine Mayer, Valerie Lamour and Pan Chan for fruitful discussions. We thank Yaëlle Wormser for her help with bacterial cultures for microscopy studies. E.Y. acknowledges a PhD fellowship from the Médicament, Toxicologie, Chimie et Imageries PhD school (MTCI, ED 563), Université Paris Cité. The PhD fellowship of J.P. acknowledges a PhD fellowship from the AMX program from École Polytechnique.

Funding: This study was supported by the IdEx Université Paris Cité, ANR-18-IDEX-0001 (S.P.), ANR-21-CE11-0003 (A.M.W.), 10.13039/501100002915Fondation pour la Recherche Médicale (grant number EQU202303016284 to P.M.A.), by the 10.13039/501100004794Centre National de la Recherche Scientifique (CNRS), 10.13039/501100003762Institut Pasteur, Université Paris Cité, 10.13039/501100019125Sorbonne Université, 10.13039/501100001677Inserm, 10.13039/100015872Université de Lille, and Institut Pasteur de Lille.

## Author contributions

A.G., N.W., and S.P. conceptualized and designed experiments; S.P. supervised the studies; A.Di., A.Dre., and B.G. conducted chemical synthesis; C.P. evaluated physicochemical properties of compounds; A.G., E.Y., and E.C. provided purified proteins; K.D. evaluated minimal inhibitory concentrations of compounds on ESKAPE strains; E.S. evaluated minimal inhibitory concentrations of *Mtb* strains; E.C. evaluated inhibitory potency of compounds on *Mtb* and *E. coli* gyrases; E.Y. and E.L. optimized microscopy conditions; J.P. carried out morphology evaluation and analyzed microscopy images; A.G. characterized mechanism of inhibition and conducted cleavage assays; E.Y., F.G., and S.P. prepared cryoEM grids and collected data; A.M., E.Y., and S.P. refined models and analyzed cryoEM data; A.A., A.M.W., P.M.A., B.D., A.B., and N.W. provided scientific and strategic guidance; A.G., N.W., and S.P. wrote the original draft of the manuscript; A.G., J.P., A.M.W., and S.P. prepared figures; A.G., N.W., and S.P. revised the draft. All authors completed and edited the paper.

## Declaration of interests

The authors declare no competing financial interests.

## STAR★Methods

### Key resources table

**Table:** 

REAGENT or RESOURCE	SOURCE	IDENTIFIER
**Bacterial strains**		

*Escherichia coli*	ATCC	ATCC 25922
*Escherichia coli*	CFPL	CFPL 8137
*Escherichia coli*	CFPL	CFPL 8141
*Escherichia coli* penicillin resistant	CFPL	CFPL 8138
*Escherichia coli* penicillin and fluoroquinolone resistant	CFPL	CFPL 8157
*Escherichia coli* BLSE	CFPL	CFPL 9003
*Escherichia coli* KPC-2	CFPL	CFPL 10273
*Escherichia coli* NDM-1	CFPL	CFPL 10269
*Escherichia coli* NDM-1	CFPL	CFPL 10385
*Escherichia coli* NDM-1	CFPL	CFPL 10386
*Klebsiella pneumoniae* VIM	CFPL	CFPL 10270
*Klebsiella pneumoniae* 0XA-48	CFPL	CFPL 10272
*Klebsiella pneumoniae* KPC-2	CFPL	CFPL 10277
*Pseudomonas aeruginosa* fluoroquinolone resistant	CFPL	CFPL 8127
*Pseudomonas aeruginosa*	CFPL	CFPL 8131
*Pseudomonas aeruginosa* MDR with protein-D2 porin defect	CFPL	CFPL 8132
*Pseudomonas aeruginosa*	CFPL	CFPL 8133
*Pseudomonas aeruginosa* fluoroquinolone resistant	CFPL	CFPL 8134
*Pseudomonas aeruginosa*	CFPL	CFPL 8135
*Pseudomonas aeruginosa* MDR with protein-D2 porin defect	CFPL	CFPL 8136
*Acinetobacter baumannii* VEB-1	CFPL	CFPL 09010
*Acinetobacter baumannii* MDR	CFPL	CFPL 09011
*Acinetobacter baumannii* methicillin, kanamycin and tobramycin resistant	CFPL	CFPL 10275
*Staphylococcus aureus* methicillin, kanamycin and tobramycin resistant	CFPL	CFPL 8143
*Staphylococcus aureus* methicillin and kanamycin resistant	CFPL	CFPL 8146
*Staphylococcus aureus*	CFPL	CFPL 8147
*Staphylococcus aureus* methicillin and fluoroquinolone resistant	CFPL	CFPL 8148
*Staphylococcus aureus*	CFPL	CFPL 8149
*Staphylococcus aureus*	CFPL	CFPL 8237
*Staphylococcus aureus*	CFPL	CFPL 8238
*Staphylococcus aureus* methicillin resistant	CFPL	CFPL 8239
*Staphylococcus aureus* methicillin and fluoroquinolone resistant	CFPL	CFPL 8240
*Staphylococcus aureus* methicillin, kanamycin, tobramycin and fluoroquinolone resistant	CFPL	CFPL 8241
*Enterococcus faecium* MDR	CFPL	CFPL 09001
*Enterococcus faecium* MDR	CFPL	CFPL 09002
*Mycobacterium tuberculosis* H37Rv WT	ATCC	ATCC 27294
*Mycobacterium tuberculosis* H37Rv harboring a D461N in GyrB (selected from the parental ATCC27294 strain)	This study	Lab collection
*Mycobacterium tuberculosis* H37Rv harboring a A90V in GyrA (selected from the parental ATCC27294 strain)	This study	Lab collection
*Mycobacterium tuberculosis* H37Rv harboring a D94G in GyrA (selected from the parental ATCC27294 strain)	This study	Lab collection
E. coli Bli5 (BL21 DE3/pDIA17)	Rogé et Betton, 2005^64^	Lab collection
*Corynebacterium glutamicum*	ATCC	ATCC 13032

**Chemicals, peptides, and recombinant proteins**		

Gepotidacin	MedChemExpress	HY-16742
Ciprofloxacin	Sigma-Aldrich	17850
Trizma Base	Sigma-Aldrich	T4661
KCl	Sigma-Aldrich	P9541
NaCl	Sigma-Aldrich	S7653
Magnesium acetate	Sigma-Aldrich	M5661
KGlu	Sigma-Aldrich	G1501
EDTA	Sigma-Aldrich	E9884
ATP	Sigma-Aldrich	A7699
Adenylyl-imidodiphosphate	Sigma-Aldrich	10102547001
DTT	Sigma-Aldrich	43815
BSA	Sigma-Aldrich	A7906
SDS	Sigma-Aldrich	L3881
Spermidine	Sigma-Aldrich	S2626
Proteinase K	Sigma-Aldrich	P2308
Agarose	Sigma-Aldrich	A9539
TBE running buffer	Sigma-Aldrich	T3913
GelRed®	Sigma-Aldrich	SCT122
SYBR™ Safe	Thermofisher Scientific	S33102
Supercoiled pBR322 DNA	New England Biolabs	N3033
Relaxed pBR322 DNA	Inspiralis	R5001
FastDigest restriction enzyme *NdeI*	Thermofisher Scientific	FD0583
Protease inhibitors cOmplete Tablets, Mini EDTA-free, EASYpack	Roche	04 693 159 001
HEPES	Calbiochem	391340
MgCl_2_.6H_2_O	Merck	1.05835.0100
Imidazole	Merck	1.04716.100
Kanamycin solution	Sigma-Aldrich	K0254
Nile Red	Enzo-Life Science	ENZ-52551
Hoechst 33342	Thermofisher Scientific	62249
p-anisidine	Acros	10483 2500
Diethyl ethoxymethylenemalonate	Acros	11434-1000
Eaton’s reagent	Sigma-Aldrich	380814
sodium azide	Alpha-Aesar	A11970
5-amino-2-methoxypyridine	Fluorochem	024176
triethyl orthoformate	Alpha-Aesar	A13587
2,2-dimethyl-1,3-dioxane-4,6-dione	Sigma-Aldrich	210145-25G
phosphorus oxychloride	Acros	191292500
sodium borohydride	Sigma-Aldrich	452874-25G
but-3-yn-1-amine	Fluorochem	F235851
1,4-benzodioxane-6-carboxaldehyde	Sigma-Aldrich	264598-5G
4-fluoro-3-nitrobenzaldehyde	Alpha-Aesar	H26181
4-hydroxy-3-nitrobenzaldehyde	Fluorochem	018721
2,3-Dihydro-1,4-benzodioxine-6-carboxylic acid	Sigma-Aldrich	658375-5G
4-fluoro-3-nitrobenzoic acid	Alpha-Aesar	A10800
ethyl thioglycolate	Alpha-Aesar	MFCD00004874

**Deposited data**		

CryoEM structure of BDM71403-bound form of *Mtb* DNA gyrase	This study	PDB 8S70 EMD-19782
CryoEM structure of gepotidacin -bound form of *Mtb* DNA gyrase	This study	PDB 8S7K EMD-19777

**Oligonucleotides**		

ME-73b (see Table S3)	This study	Eurogentec
ME-77b (see Table S3)	This study	Eurogentec

**Recombinant DNA**		

pET28-His6-TEV-GyrBA	Petrella et al., 2019^36^	Lab collection

**Software and algorithms**		

cryoSPARC	Punjani et al., 2017^65^	https://cryosparc.com
PHENIX	Adams et al., 2010^66^	https://www.phenix-online.org/
COOT	Emsley et al., 2010^67^	https://www2.mrc-lmb.cam.ac.uk/personal/pemsley/coot/
CHIMERAX	Meng et al., 2023^68^	https://www.cgl.ucsf.edu/chimerax/
CHIMERA	Pettersen et al., 2004^69^	https://www.cgl.ucsf.edu/chimera/
Omnipose	Cutler et al., 2022^70^	https://github.com/kevinjohncutler/omnipose
Fiji 70 and plugin MicrobeJ	Ducret et al., 2016^71^	https://www.microbej.com/download-2/
GraphPad Prism 9.0	GraphPad Prism Software, Inc.	https://graphpad.com
ImageJ 1.53k	Schneider et al., 2012^72^	https://imagej.nih.gov/ij/
Topspin 4.4	Bruker	https://www.bruker.com/en/products-and-solutions/mr/nmr-software/topspin.html
MassLynx V4.1	Waters inc.	www.waters.com
MS Workstation 6.3.0	Varian	www.varian.com

**Other**		

HisTrap HP 5 mL columns	Cytiva Life Sciences	17-5248-01
Heparin HP 5 ml columns	Cytiva Life Sciences	17-0407-03
HiLoad 16/60 Superdex 200 column	Cytiva Life Sciences	17-1069-01
Superose 6 Increase 5/150 GL	Cytiva Life Sciences	29-0915-97
Vivaspin 20 concentrators (MWCO 100 kDa)	Sartorius	VS2041
Quantifoil copper R2/2 200 mesh grids (lot 233324)	Quantifoil	N1-C16nCu20-01

### Experimental model and study participant details

#### Bacterial culture

All strains listed in the key resources table. American Type Culture Collection strains (reference strains from the ATCC), and drug multi-resistant clinical strains (that reflect what is encountered in clinical cases from the CHRU Lille, France).^73^ For each strain was added the registration number in the CFPL (collection of the faculty of pharmacy of Lille - “*collection de la Faculté de Pharmacie de Lille”*), followed by the mentions of antimicrobial resistance or the type of enzymes implicated in resistance. Species identification (determined by mass spectrometry) and antibiogram of each bacterium (cultivated or cryopreserved) are evaluated three times per year. ESKAPE strains have been grown in Brain Heart Infusion medium at 37°C, *Mtb* strains have been grown in 7H11 agar supplemented with 10% Middlebrook OADC.

### Method details

#### Compounds

Gepotidacin was purchased from MedChemExpress. Ciprofloxacin and all other chemicals were purchased from Sigma. All inhibitors were solubilized as recommended by suppliers. Synthesized molecules from our series were all resuspended at 50 mM in 100% DMSO.

#### Antibacterial activity measurement

The various selected ESKAPE microorganisms were all able to grow aerobically in Mueller Hinton Agar (MHA) media. Standardized methodology with internationally recognized protocols (CLSI, 2006) were used to determine MIC. MIC determinations were carried out using the agar dilution method for 37 bacterial strains by diluting the compounds in MHA media. The inhibitory concentrations ranged between 0.0625 and 64 μg/ml in eleven 2-fold dilutions (64, 32, 16, 8, 4, 2, 1, 0.5, 0.25, 0.125, 0.0625 μg/ml), Petri dishes (Controls and BDM71403 (**10a**)), were inoculated with strains (10^4^ CFU, obtained by dilution in brain heart) using a Steer's replicator and were incubated at 37°C for 24 h. MIC was defined as the lowest concentration of extract without bacterial growth after incubation. The MIC determination against wild-type and fluoroquinolones resistant *Mtb* H37Rv were determined by agar dilution method on 7H11 agar supplemented with 10% Middlebrook OADC (oleic acid, albumin, dextrose and catalase). The 1 μL of a McFarland 0.5 turbidity standard suspension was inoculated with a MAST URI®DOT multipoint inoculator delivering approximately 10^5^ CFU per spot. Colonies were enumerated after 21 to 30 days of incubation at 37°C. The MIC was defined as the lowest concentration of antibiotic resulting in complete inhibition of growth or in growth of fewer than 10 colonies (<1% of the inoculum).^74^

#### Protein production and purification

Expression and purification of *Mtb* fusion-protein (GyrBA) or non-fusion proteins (GyrB, GyrA, and mutants D461A, A90V and D94G) were conducted as previously described.^36^ In brief, *E. coli* Bli5 (DE3)^64^ were transformed with pET-28a constructs harboring each ORF of interest, and protein expression with an N-terminus His tag was induced *via* a lactose-driven autoinduction. After an overnight culture at 30°C, bacteria were harvested by centrifugation and resuspended in buffer A (20 mM Tris-HCl, pH8, 500 mM NaCl, 10 mM imidazole, 1 mM DTT, 5 mM MgCl_2_, 5% Glycerol) supplemented with a cOmplete™ ULTRA EDTA-free protease inhibitor tablet (Roche Diagnostics). Bacterial lysis was conducted on a Cell Disruption Lysis System. Proteins were then purified from the soluble extract on a Ni^2+^-affinity column (HisTrap HP column, Cytiva) with a linear imidazole gradient. Fractions of interest were subjected to TEV digestion in a dialysis buffer (10 mM Tris-HCl pH 7.5, 80 mM NaCl, 1 mM DTT, 1 mM EDTA and 10% glycerol). Untagged proteins were then collected after a passage on a Heparin column (Heparin HP column, Cytiva) with a linear NaCl gradient (80 to 1 000 mM) and a size-exclusion chromatography (Superdex 200 column, Cytiva) in buffer B (20 mM Tris-HCl pH 7.5, 100 mM KCl, 1 mM DTT, 1 mM EDTA, 10% glycerol). Proteins were fast frozen in liquid nitrogen and stocked at -80°C. Protein concentration was determined by using the molar absorption coefficient predicted from the amino acid sequence by the ProtParam tool.^75^

#### DNA supercoiling inhibition

Gyrase inhibition evaluation of each synthesized molecule were effectuated *via* a DNA supercoiling assay as described previously.^76^ Briefly, reactions were carried out by adding 100 ng GyrA and 200 ng GyrB in the presence of 300 ng of relaxed pBR322 plasmid DNA (Inspiralis) and 1 mM ATP in a reaction buffer containing 40 mM Tris-HCl pH 7.5, 25 mM KCl, 2 mM spermidine, 4 mM DTT, 0.1 mM EDTA, 6 mM magnesium acetate and 100 mM potassium glutamate. Reactions were conducted for one hour at 37°C.

#### DNA cleavage

DNA cleavage assay was conducted in a final volume of 30 μL containing 40 mM Tris-HCl pH 7.5, 25 mM KCl, 2 mM spermidine, 4 mM DTT, 0.1 mM EDTA, 6 mM of magnesium acetate, 5 nM of supercoiled pBR322 plasmid DNA (New England Biolabs), and 2 mM ATP (Sigma), in the absence or presence of each inhibitor. Reactions were launched for one hour at 37°C by addition of 200 nM of *Mtb* GyrBA to the reaction mixture. To liberate DNA plasmid from gyrase, 0.1 mg/mL of proteinase K and 0.2% SDS were added to the reaction mixture. After incubation for one hour at 37°C, samples were then supplemented with loading dye (50% glycerol and 0.025% bromophenol blue) and were analyzed by migration on 1% agarose gel in 1X TAE Buffer in the presence of 0.5 μg/mL of SYBR™ Safe. For verification of cleavage persistence, assays were adapted as previously described.^37^^,^^38^^,^^39^ Briefly, mixtures in the presence of 25 μM gepotidacin or 25 μM BDM71403 in a total volume of 30 μL contain the same composition as cleavage assay but in the absence of ATP, and in the presence of higher concentrations of *Mtb* GyrBA (1 μM final concentration) and pBR322 plasmid DNA (100 nM final concentration). After one hour at 37°C, mixtures were diluted 20-fold with reaction buffer and incubated again at 37°C. Aliquots of 30 μL were taken at different time frames (from 0 to 6 hours) and were then treated with proteinase K and SDS and analyzed as for cleavage assays. Linearized DNA used for reference was generated by digestion of supercoiled pBR322 plasmid DNA by FastDigest restriction enzyme *NdeI* (Thermofisher Scientific). Control relaxed DNA was purchased from Inspiralis. Bands of interest were quantified with ImageJ Software^72^ and represented using GraphPad Prism 9.0 (GraphPad Prism Software, Inc.).

#### Cryo-electron microscopy studies

##### Nucleic acid preparation

A 150bp DNA duplex was reconstituted using two phosphorylated asymmetric synthetic oligonucleotides obtained from Eurogentec. Oligonucleotides ME-73b, ME-77b and their corresponding complementary strands (5’-3’ sequences in Table S3) were dissolved in DNase-free water at 1 mM concentration. The 150 bp double stranded DNA was assembled by mixing at 1:1 molar ratio for each oligonucleotide, annealed by incubating at 95°C for 2 min and then decreasing the temperature by 1°C every 1 min until reaching 20°C.

##### Nucleoprotein complex formation for cryoEM

The purified *Mtb* GyrBA was mixed with the 150 bp dsDNA at 1:1 molar ratio with a final concentration of 2 μM. GyrBA, DNA and inhibitors mixtures at 40 μM were incubated for 10 min at 37°C. Adenylyl-imidodiphosphate (Sigma) was then added to the ternary complex at 25 μM and further incubated for 30 min at 30°C. The complex was than stored at 4°C until sample freezing on cryoEM grids.

##### CryoEM sample preparation

Quantifoil copper R2/2 200 mesh grids were glow-discharged using a Solarus II plasmacleaner (Gatan, Inc), for 10 sec at 5W prior to the application of 4 μL of the complex. The grids were plunge-frozen in liquid ethane using a Vitrobot Mk-IV (Thermo Fisher Scientific) set at 8°C and 100% humidity, blot time 3s and blot force +20.

##### CryoEM data collection

CryoEM imaging was performed on a Titan Krios microscope (FEI) (EMBL, Heidelberg for BDM71403-bound complex and Nanoimaging facility, Institut Pasteur, Paris for gepotidacin-bound complex) operated at 300 kV equipped with a Quantum-K3 camera (Gatan) and a GIF Quantum energy filter (Gatan). For the BDM71403-bound complex, images were recorded in EFTEM nanoprobe mode with Serial EM 50 in super-resolution counting mode with a pixel size of 0.645 Å and a constant defocus target of - 520 nm. Four datasets were collected with a total dose of 49 e^-^/Å^2^ distributed on 40 frames. A total of 12,888 movies were recorded (Table S2). For the gepotidacin-bound complex, images were recorded in a counted mode with the software EPU and a pixel size of 0.860 Å. A total of 7.900 movies were recorded (Table S2).

##### CryoEM and image processing

All image processing steps were done using CryoSPARC.^65^ Movies motion correction was performed using *Patch Motion Correction*. The contrast transfer function (CTF) parameters were estimated using *Patch CTF estimation*. A first particle picking done with the *blob picker* tool was used to generate 2D classes that were subsequently used as templates for template-based particles picking. Particles from 2D classes displaying high-resolution features were selected and used to generate a first reference-free 3D *ab initio* model. The particles were further classified in 3D using *Heterogenous Refinement.* After particles classification, Non-Uniform (NU) refinement without imposing any symmetry was performed, resulting in a final reconstruction at ∼ 2.8 Å and ∼ 3.1 Å resolution, for BDM71403- and gepotidacin-bound structures, respectively.

##### Model building and refinement

In an initial step, we fitted the crystal structure of *Mtb* gyrase cleavage core (RCSB Protein Data Bank (PDB) code: 5BS8) after cleaning the double-cleaved strands DNA and the two moxifloxacin molecules, into our map. We manually built the double-strand DNA using Coot^77^ and placed the molecules in the corresponding density. Several cycles of refinement were done using Phenix.^78^^,^^79^

#### Phase contrast, fluorescence microscopy and image analysis

For imaging, a culture of *Cglu* ATCC13032 was grown overnight in minimal medium CGXII supplemented with 4% sucrose, diluted to OD600=1 the following day until the early exponential phase (5h) before adding the antibiotic to the cultures at a concentration equivalent to 10x MIC value for each antibiotic. After 24h of growth, the cultures were collected for imaging. For membrane and DNA staining, Nile Red (Enzo Life Sciences) and Hoechst 33342 (Thermofisher Scientific) were added to the culture (1.6 μg/ml and 2 μg/ml final concentration respectively) prior to placing them on 2% agarose pads prepared with minimal medium CGXII. Cells were visualized using a Zeiss Axio Observer Z1 microscope fitted with an Orca Flash 4 V2 sCMOS camera (Hamamatsu) and a Pln-Apo 63X/1.4 oil Ph3 objective. Images were collected with Zen Blue 2.6 (Zeiss). They were segmented using a specifically trained version of Omnipose,^70^ the software Fiji 70 and the plugin MicrobeJ version 5.13o^71^ to generate violin plots. The experiments were performed as biological triplicates. For statistical analysis, due to the important number of cells analyzed in each sample, Cohen’s *d* value was used to describe effect sizes between different strains independently of sample size:

$$d=\frac{{\text{mean}}_{2}-\;{\text{mean}}_{1}}{\sqrt{\frac{\left(n_{1}-1\right)*{{\text{SD}}_{1}}^{2}+\left(n_{2}-1\right)*{{\text{SD}}_{2}}^{2}}{n_{1}+n_{2}-2}}}$$

Values were interpreted according to the intervals of reference suggested by Cohen^80^ and expanded by Sawilowsky,^81^ as follows: small (n.s.), *d* < 0.50; medium (∗), 0.50 < *d* < 0.80; large (∗∗), 0.80 < *d* <1.20; very large (∗∗∗), 1.20 < *d* < 2.0; huge (∗∗∗∗), *d* > 2.0.

#### Chemistry

All commercial reagents and solvents were used without further purification. HPLC-MS analysis was performed on a Waters Alliance Micromass ZQ 2000 using a C_18_ TSK-GEL Super ODS 2 μm particle size column, dimensions 50 mm × 4.6 mm. A gradient starting from 100% H_2_O/0.1% formic acid and reaching 20% H_2_O/80% CH_3_CN/0.08% formic acid within 10 minutes at a flow rate of 1 mL/min was used. High-resolution mass spectra were recorded on a HPLC-MS-TOF, Waters LCT Premier XE Micromass, using a C_18_ X-Bridge 3.5 μm particle size column, dimensions 50 mm × 4.6 mm. A gradient starting from 98% H_2_O 5 mM ammonium formate pH=3.8 and reaching 100% CH_3_CN 5 mM ammonium formate pH 3.8 within 3 minutes at a flow rate of 1 mL/min was used. NMR spectra were recorded on a Bruker DRX-300 spectrometer (Data S1). The results were calibrated to signals from the solvent as an internal reference [*e.g.* 7.26 (residual CDCl_3_) and 77.16 (CDCl_3_) ppm, 2.50 (residual DMSO-d^6^) and 39.52 (DMSO-d^6^) ppm for ^1^H and ^13^C NMR spectra, respectively]. Chemical shifts (δ) are in parts per million (ppm) downfield from tetramethylsilane (TMS). The assignments were made using one-dimensional (1D) ^1^H and ^13^C spectra and two-dimensional (2D) HSQC-DEPT, COSY and HMBC spectra. NMR coupling constants (J) are reported in Hertz (Hz), and splitting patterns are indicated as follows: s (singlet), brs (broad singlet), d (doublet), dd (doublet of doublet), ddd (double of doublet of doublet), dt (doublet of triplet), t (triplet), td (triplet of doublet), q (quartet), m (multiplet).

##### General procedures for the synthesis of azides and alkynes required for click reactions

Compounds of interest were synthesized as follows:

*4-Hydroxy-6-methoxy-quinoline-3-carboxylic acid ethyl ester (1).* A mixture of *p*-anisidine (1.23 g, 10.00 mmol) and diethylethoxymethylenemalonate (2.39 g, 11.00 mmol) in toluene (10 mL) was heated under reflux for 3 h and then evaporated under reduced pressure. 15 mL of Eaton’s reagent were added to the residue and the mixture was heated at 90°C for 18 h then cooled to 5°C and slowly transferred to a saturated sodium carbonate solution (100 mL) that was cooled to 10°C. The solid was filtered, washed with water (100 mL) and dried under vacuum to afford compound 1 as a beige powder (1.71 g, 70%). LC-MS (EI(+)): m/z 248 [M+H]^+^; RT=1.73 min. NMR ^1^H (DMSO-d_6_, 300 MHz): δ ppm 8.50 (s, 1H), 7.59-7.54 (m, 2H), 7.28 (d, *J* = 8.9 Hz, 1H), 4.20 (q, *J* = 7.2 Hz, 2H), 3.83 (s, 3H), 1.27 (t, *J* = 6.9 Hz, 3H).

*4-Hydroxy-6-methoxy-quinoline-3-carboxylic acid (2).* A mixture of compound 1 (1.71 g, 6.95 mmol) and sodium hydroxide 2N was stirred at room temperature for 48 h. Hydrochloric acid 2N was added until precipitate was formed. The precipitate was filtered, washed with water and dried under vacuum to afford compound 2 as a beige powder (1.48 g, 98%). LC-MS (EI(+)): m/z 220 [M+H]^+^; RT=1.85 min. NMR ^1^H (DMSO-d_6_, 300 MHz): δ ppm 13.41 (s, 1H), 8.82 (s, 1H), 7.88-7.41 (m, 3H), 3.90 (s, 3H).

*4-Hydroxy-6-methoxy-quinoline (****3a****).* A mixture of compound 2 (1.48 g, 6.75 mmol) and diphenylether was heated under reflux (250°C) for 30 min then cooled to room temperature, to which heptane was added. Compound **3a** (1.11 g, 94%) was obtained by filtration and washing with heptane and ethyl acetate. LC-MS (EI(+)): m/z 176 [M+H]^+^; RT=1.48 min. NMR ^1^H (DMSO-d_6_, 300 MHz): δ ppm 11.85 (s, 1H), 7.84 (d, *J* = 7.5 Hz, 1H), 7.53 (d, *J* = 9.0 Hz, 1H), 7.49 (d, *J* = 3.0 Hz, 1H), 7.28 (dd, *J* = 3.0, 9.0 Hz, 1H), 6.00 (d, *J* = 7.2 Hz, 1H), 3.82 (s, 3H).

*4-Chloro-6-methoxy-quinoline (****4a****).* A mixture of compound **3a** (1.11 g, 6.30 mmol) and phosphorus oxychloride (4 mL) was stirred at 110°C for 1 h. The excess of phosphorus oxychloride was removed under reduced pressure. The residue was quenched into crushed ice and neutralized using saturated solution of sodium bicarbonate. The solid was filtered and dried under vacuum to afford compound **4a** (1.03 g, 84%) as a brown powder. LC-MS (EI(+)): m/z 194 [M+H]^+^; RT=2.55 min. NMR ^1^H (DMSO-d_6_, 300 MHz): δ ppm 8.68 (d, *J* = 4.5 Hz, 1H), 8.02 (d, *J* = 9.0 Hz, 1H), 7.73 (d, *J* = 4.5 Hz, 1H), 7.52 (dd, *J* = 2.7, 9.3 Hz, 1H), 7.44 (d, *J* = 2.4 Hz, 1H), 3.96 (s, 3H).

*4-Azido-6-methoxy-quinoline (****5a****).* To a suspension of compound **4a** (1.03 g, 5.30 mmol) in ethanol/water 1/1 (25 mL), sodium azide (1.23 g, 26.60 mmol) was added and the reaction mixture was stirred at 100°C for 40 h then cooled to room temperature and quenched with cold water. The solid was filtered, washed with water and dried under vacuum to afford compound **5a** (989 mg, 93%) as a brown powder. LC-MS (EI(+)): m/z 173 [M-N_2_+H]^+^; RT=2.32 min. NMR ^1^H (DMSO-d_6_, 300 MHz): δ ppm 8.69 (d, *J* = 3.3 Hz, 1H), 7.92 (d, *J* = 8.7 Hz, 1H), 7.55-7.20 (m, 3H), 3.90 (s, 3H). NMR ^13^C (DMSO-d_6_, 75 MHz): δ ppm 157.4, 147.9, 144.5, 143.8, 130.6, 122.9, 121.7, 110.0, 99.8, 55.5.

*5-[(6-Methoxy-pyridin-3-ylamino)-methylene]-2,2-dimethyl-[1,3]dioxane-4,6-dione (6).* To a solution of 5-amino-2-methoxypyridine (1.24 g, 10.00 mmol) in ethanol (10 mL) was added triethyl orthoformate (1.66 mL, 10.00 mmol) and 2,2-dimethyl-1,3-dioxane-4,6-dione (Meldrum’s acid) (1.44 g, 10.00 mmol). The mixture was heated under reflux for 4 h then cooled to room temperature. The resulting suspension was filtered, washed with ethanol and dried under vacuum to afford the compound 6 as a beige powder (2.28 g, 82%). LC-MS (EI(+)): m/z 279 [M+H]^+^; RT=2.15 min. NMR ^1^H (DMSO, 300 MHz): δ ppm 11.25 (s, 1H), 8.45 (s, 1H), 8.38, (d, *J* = 2.7 Hz, 1H), 7.97 (dd, *J* = 3.0, 9.0 Hz, 1H), 6.88 (d, *J* = 9.0 Hz, 1H), 3.86 (s, 3H), 1.67 (s, 3H).

*6-Methoxy-[1,5]naphthyridin-4-ol (****3b****).* A suspension of compound 6 (2.18 g, 7.83 mmol) in 10 mL diphenyl ether was added in small portions to boiling diphenyl ether (10 mL). The mixture was stirred at 250°C until gas evolution was ceased (4 min after completion of addition), then cooled, diluted with heptane and filtered. The precipitate was washed with heptane and ethyl acetate and dried under vacuum to afford the compound **3b** (1.25 g, 91%) as a beige powder. LC-MS (EI(+)): m/z 177 [M+H]^+^; RT=1.23 min. NMR ^1^H (DMSO-d_6_, 300 MHz): δ ppm 11.88 (s, 1H), 8.13 (d, *J* = 7.5 Hz, 1H), 7.80 (d, *J* = 9.0 Hz, 1H), 7.17 (d, *J* = 7.5 Hz, 1H), 6.22 (d, *J* = 9.0 Hz, 1H), 3.92 (s, 3H).

*8-Chloro-2-methoxy-[1,5]naphthyridine (****4b****).* A mixture of compound **3b** (1.15 g, 6.52 mmol) and phosphorus oxychloride (7 mL) was stirred at 110°C for 1.5 h. The excess of phosphorus oxychloride was removed under reduced pressure. The residue was quenched into crushed ice and neutralized using saturated solution of sodium bicarbonate. The solid was filtered and dried under vacuum to afford compound **4b** (1.02 g, 80%) as a brown powder. LC-MS (EI(+)): m/z 195 [M+H]^+^; RT=2.56 min. NMR ^1^H (DMSO-d_6_, 300 MHz): δ ppm 8.57 (s, d, *J* = 4.7 Hz, 1H), 8.27, (d, *J* = 9.0 Hz, 1H), 7.05 (d, *J* = 4.7 Hz, 1H), 7.30 (d, *J* = 9.0 Hz, 1H), 4.04 (s, 3H).

*8-Azido-2-methoxy-[1,5]naphthyridine (****5b****).* To a suspension of compound **4b** (998 mg, 5.13 mmol) in ethanol/water 1/1 (20 mL), sodium azide (2.5 g, 38.46 mmol) was added and the reaction mixture was stirred at 100°C for 7 days then cooled to room temperature and quenched with cold water. The solid was filtered, washed with water and dried under vacuum to afford compound **5b** (877 mg, 85%) as a red-brown powder. LC-MS (EI(+)): m/z 174 [M-N_2_+H]^+^; RT=2.58 min. NMR ^1^H (DMSO-d_6_, 300 MHz): δ ppm 8.62 (d, *J* = 4.8 Hz, 1H), 8.28 (d, *J* = 9.0 Hz, 1H), 7.33 (d, *J* = 9.0 Hz, 1H), 7.20 (d, *J* = 4.8 Hz, 1H) 3.99 (s, 3H).

##### General procedures for the synthesis of aryl-ynil-amines

Aldehyde (0.50 mmol, 1.0 eq) was dissolved in a mixture of methanol/chloroform 1:1 (2 mL). Molecular sieves 4Å and amine (0.60 mmol, 1.2 eq) were added and the mixture was stirred at 60°C until the imine formation reached completion (the reaction was followed by NMR ^1^H). Then, sodium borohydride (0.50 mmol, 1.0 eq) was added at 0°C and the mixture was warmed to room temperature and stirred for 18 h. Molecular sieves were filtered and chloroform (20 mL) was added to the filtrate. The organic layer was washed with saturated sodium bicarbonate solution, brine and hydrochloric acid 1M. The aqueous layer was basified with sodium hydroxide 2M and extracted with chloroform (20 mL x 3). The combined organic layers were washed with brine, dried over anhydrous magnesium sulfate and concentrated under reduced pressure to afford the desired product.

*6-((But-3-ynylamino)methyl)-2H-benzo[b][1,4]thiazin-3(4H)-one (****7a****)* was prepared following the general procedure using aldehyde 3-oxo-3,4-dihydro-2H-benzo[1,4]thiazine-6-carbaldehyde and but-3-yn-1-amine to afford 96.4 mg (77%) of a yellow solid. NMR ^1^H (CDCl_3_, 300 MHz): δ ppm 9.62 ( br s, 1H), 7.23 (d, *J* = 8.1 Hz, 1H), 6.99-6.89 (m, 2H), 3.75 (s, 2H), 3.41 (s, 2H), 2.77 (t, *J* = 6.6 Hz, 2H), 2.39 (td, *J* = 2.7, 6.6 Hz, 2H), 2.00 (t, *J* = 2.7 Hz, 1H).

*N-((2,3-Dihydrobenzo[b][1,4]dioxin-6-yl)methyl)but-3-yn-1-amine (****7b****)* was prepared following the general procedure using 1,4-benzodioxane-6-carboxaldehyde and but-3-yn-1-amine to afford 103.6 mg (95%) of a yellow oil. NMR ^1^H (CDCl_3_, 300 MHz): δ ppm 6.87-6.80 (m, 3H), 4.26 (s, 4H), 3.70 (s, 2H), 2.80 (t, *J* = 6.6 Hz, 2H), 2.42 (td, *J* = 2.7, 6.6 Hz, 2H), 2.01 (t, *J* = 2.7 Hz, 1H).

*6-But-3-ynylaminomethyl-4H-benzo[1,4]oxazin-3-one (****7c****)* was prepared following the general procedure using 3-oxo-3,4-dihydro-2H-benzo[1,4]oxazine-6-carbaldehyde and but-3-yn-1-amine to afford 85.1 mg (74%) of a yellow solid. NMR ^1^H (CDCl_3_, 300 MHz): δ ppm 9.27 (br s, 1H), 6.94-6.83 (m, 3H), 4.60 (s, 2H), 3.73 (s, 2H), 2.78 (t, *J* = 6.6 Hz, 2H), 2.40 (td, *J* = 2.7, 6.6 Hz, 2H), 2.01 (t, *J* = 2.7 Hz, 1H).

The amines and aldehydes required for the above-mentioned reactions were prepared as follows:

###### 3-Oxo-3,4-dihydro-2H-benzo[1,4]thiazine-6-carbaldehyde

To a solution of 4-fluoro-3-nitro-benzaldehyde (793 mg, 4.69 mmol) in DCM (20 mL) at 0°C was added triethylamine (784 μL, 5.63 mmol) and ethyl thioglycolate (519 μL, 4.74 mmol). The mixture was warmed to room temperature and stirred for 7 h then water and dichloromethane were added. The organic layer was separated and washed with hydrochloric acid 1M, brine, then dried over anhydrous magnesium sulfate and concentrated under reduced pressure to provide (4-formyl-2-nitro-phenylsulfanyl)-acetic acid ethyl ester. This intermediate was dissolved in acetic acid (25 mL), iron powder (2.62 g, 46.90 mmol) was added and the resulting suspension was heated to 60°C and stirred for 1 h. The reaction mixture was cooled to room temperature and filtered through celite. The filtrate was partitioned between water and ethyl acetate and the organic layer was washed with saturated sodium bicarbonate solution and brine, dried over anhydrous magnesium sulfate and evaporated to afford the desired aldehyde (900 mg, 99%) as a yellow solid.

NMR ^1^H (DMSO-d_6_, 300 MHz): δ ppm 10.39 (br s, 1H), 9.90 (s, 1H), 7.59-7.42 (m, 3H), 3.57 (s, 2H).

###### 3-Oxo-3,4-dihydro-2H-benzo[1,4]oxazine-6-carbaldehyde

To a solution of 4-hydroxy-3-nitro-benzaldehyde (334.2 mg, 2.00 mmol) in DMF (4 mL) was added potassium carbonate followed by ethyl chloroacetate (321.0 μL, 3.00 mmol) dropwise. The reaction mixture was stirred at 50°C for 80 h, then warmed to room temperature, diluted with water and extracted with ethyl acetate. The combined organic layers were dried over anhydrous magnesium sulfate and concentrated under reduced pressure to afford (4-formyl-2-nitro-phenoxy)-acetic acid ethyl ester, which was dissolved in acetic acid (15 mL). Thereafter, iron powder (1.10 g, 20.00 mmol) was added and the resulting suspension was stirred at 65°C for 4h. The reaction mixture was cooled to room temperature and filtered through celite. The filtrate was partitioned between water and ethyl acetate and the organic layer was washed with saturated sodium bicarbonate solution and brine, dried over anhydrous magnesium sulfate and evaporated to afford the desired aldehyde (186.0 mg, 54%) as a beige solid. NMR ^1^H (DMSO-d_6_, 300 MHz): δ ppm 10.98 (br s, 1H), 9.84 (s, 1H), 7.54 (dd, *J* = 1.8, 8.4 Hz, 1H), 7.38(d, *J* = 1.8 Hz, 1H), 7.14 (d, *J* = 8.4 Hz, 1H), 4.72 (s, 2H).

##### General procedure for the synthesis of aryl-ynil-amides

Carboxylic acid (0.50 mmol, 1.0 eq.) was dissolved in ethyl acetate (2 mL). *N*,*N*-diisopropylethylamine (1.50 mmol, 3.0 eq.) and T3P 50% solution in ethyl acetate (0.80 mmol, 1.6 eq.) were added in order to activate the carboxylic acid. Amine was added and the mixture was stirred at 50°C until the reaction reached completion then was washed twice with 1N hydrochloric acid, twice with saturated sodium bicarbonate solution and once with brine. The organic layer was dried over anhydrous magnesium sulfate and evaporated under reduced pressure to afford the desired amide.

*N-but-3-ynyl-3-oxo-4H-1,4-benzothiazine-6-carboxamide* (**8a**) was prepared following the general procedure using 3-Oxo-3,4-dihydro-2H-benzo[1,4]thiazine-6-carboxylic acid and but-3-yn-1-amine to afford 95.2 mg (73%) of a beige solid. NMR ^1^H (DMSO-d_6_, 300 MHz): δ ppm 10.69 (s, 1H), 8.60 (t, *J* = 5.4 Hz, 1H), 7.47-7.40 (m, 3H), 3.50 (s, 2H), 3.32 (td, *J* = 5.4, 7.2 Hz, 2H), 2.83 (t, *J* = 2.4 Hz, 1H), 2.40 (td, *J* = 2.4, 7.2 Hz, 2H).

*N-but-3-ynyl-2,3-dihydro-1,4-benzodioxine-6-carboxamide* (**8b**) was prepared following the general procedure using 2,3-Dihydro-1,4-benzodioxine-6-carboxylic acid and but-3-yn-1-amine to afford 93.5 mg (81%) of a beige solid. NMR ^1^H (DMSO-d_6_, 300 MHz): δ ppm 8.48 (t, *J* = 5.4 Hz, 1H), 7.37-7.34 (m, 2H), 6.91 (d, *J* = 8.4 Hz, 1H), 4.27 (s, 4H), 3.37-3.31 (m, 2H), 2.82 (t, *J* = 2.7 Hz, 1H), 2.40 (td, *J* = 2.7, 7.2 Hz, 2H).

*3-oxo-4H-1,4-benzothiazine-6-carboxylic acid* required for the above-mentioned reaction was prepared as follows:

*4-[(2-ethoxy-2-oxoethyl)thio]-3-nitrobenzoic acid*: to a solution of 4-fluoro-3-nitrobenzoic acid (1.85 g, 10.00 mmol) in dichloromethane (30 mL) at 0°C was added triethylamine (2.79 mL, 20.00 mmol) and ethyl thioglycolate (1.21 mL, 11 mmol). The mixture was warmed to room temperature and stirred for 70 h then water and dichloromethane were added. A precipitate appeared which was filtered and dried to give the desired compound (2.77 g, 97%). NMR ^1^H (CD_2_Cl_2_, 300 MHz): δ ppm 8.91 (d, *J* = 1.8 Hz, 1H), 8.25 (dd, *J* = 2.1, 8.4 Hz, 1H), 7.61 (d, *J* = 8.4 Hz, 1H), 4.22 (q, *J* = 7.2 Hz, 2H), 3.86 (s, 2H), 1.27 (t, *J* = 7.2 Hz, 3H).

*Ethyl 4-[(2-ethoxy-2-oxoethyl)thio]-3-nitrobenzoate*: to a solution of compound 4-[(2-ethoxy-2-oxoethyl)thio]-3-nitrobenzoic acid (1.50 g, 5.30 mmol) in ethanol was added sulfuric acid (2.5 mL) and the mixture was stirred at 80°C for 18 h. The reaction mixture was cooled to room temperature and water was added. The product was extracted with ethyl acetate. The extract was washed with brine, dried over anhydrous magnesium sulfate and evaporated under reduced pressure to afford the desired compound (1.65 g, qt). NMR ^1^H (CD_2_Cl_2_, 300 MHz): δ ppm 8.83 (d, *J* = 1.8 Hz, 1H), 8.19 (dd, *J* = 1.8, 8.7 Hz, 1H), 7.56 (d, *J* = 8.7 Hz, 1H), 4.40 (q, *J* = 6.9 Hz, 2H), 4.21 (q, *J* = 7.2 Hz, 2H), 3.83 (s, 2H), 1.40 (t, *J* = 7.2 Hz, 3H), 1.26 (t, *J* = 6.9 Hz, 3H).

*ethyl 3-oxo-4H-1,4-benzothiazine-6-carboxylate* : to a solution of ethyl 4-[(2-ethoxy-2-oxoethyl)thio]-3-nitrobenzoate (1.60 g, 5.10 mmol) in acetic acid (30 mL) ) was added iron powder (2.80 g, 51.00 mmol) and the resulting suspension was stirred at 60°C for 1 h. The reaction mixture was cooled to room temperature and filtered through celite. The filtrate was partitioned between water and ethyl acetate and the organic layer was washed with saturated sodium bicarbonate solution and brine, dried over anhydrous magnesium sulfate and evaporated to afford the desired compound (830 mg, 69%). NMR ^1^H (CD_2_Cl_2_, 300 MHz): δ ppm 8.33 (br s, 1H), 7.66 (dd, *J* = 1.8, 8.4 Hz, 1H), 7.52 (d, *J* = 1.8 Hz, 1H), 7.40 (d, *J* = 8.4 Hz, 1H), 4.35 (q, *J* = 6.9 Hz, 2H), 3.47 (s, 2H), 1.37 (t, *J* = 6.9 Hz, 3H).

*3-oxo-4H-1,4-benzothiazine-6-carboxylic acid* : to a solution of ethyl 3-oxo-4H-1,4-benzothiazine-6-carboxylate (830 mg, 3.50 mmol) in THF (5 mL), was added 1N sodium hydroxide (17 mL) and the mixture was stirred at room temperature for 5 days. 1N hydrochloric acid was added and the resulting precipitate was filtered, washed with water and dried to give the desired carboxylic acid (638 mg, 87%) as a beige solid. NMR ^1^H (DMSO-d_6_, 300 MHz): δ ppm 12.99 (br s, 1H), 10.73 (br s, 1H), 7.59-7.39 (m, 3H), 3.53 (s, 3H).

##### General procedures for click-chemistry reactions

###### Procedure A

Azide (1.0 eq.) and alkyne (1.0 eq.) were mixed in *t*-BuOH/water (1:1) to form a 0.1 M solution. Sodium ascorbate (0.4 eq.) and copper acetate monohydrate (0.2 eq.) were added and the mixture was stirred at 50°C overnight. After cooling at room temperature, the product was extracted thrice with chloroform and washed with brine. The aqueous layers were re-extracted with chloroform and ethyl acetate. All the organic layers were combined, dried over anhydrous magnesium sulfate and evaporated under reduced pressure. The resulting crude product was purified by flash-chromatography on silica gel using a mixture of dichloromethane and methanol as eluent.

###### Procedure B

Azide (1.0 eq.) and alkyne (1.0 eq.) were mixed in *t*-BuOH/water (1:1) to form a 0.1M solution. Sodium ascorbate (0.4 eq.) and copper acetate monohydrate (0.2 eq.) were added and the mixture was stirred at 50°C overnight. The reaction mixture was diluted with water (10 mL), cooled in ice, and the precipitate formed was filtered, washed with water and dried under vacuum.

###### Procedure C

Azide (1.0 eq., 0.10 mmol) and alkyne (1.0 eq., 0.10 mmol) were mixed in 2 mL of water. The reaction mixture was stirred at 110°C for 24 h. Water was evaporated and the residue was purified by flash-chromatography on silica gel using a mixture of dichloromethane and methanol as eluent.

*BDM71368 (****9a****)* was prepared following the general procedure A using azide **5a** (26.8 mg, 0.134 mmol) and alkyne 7a (33.0 mg, 0.134 mmol). The crude product was purified by flash-chromatography on silica gel DCM/MeOH 98:2, to give a brown oil (17.0 mg, 28%). Purity > 95%; LC-MS (EI(+)): m/z 447 [M+H]+; RT=1.90 min; NMR 1H (DMSO-d_6_, 300 MHz): δ ppm 10.53 (br s, 1H), 8.92 (d, J = 4.5 Hz, 1H), 8.59 (s, 1H), 8.11 (d, J = 9.3 Hz, 1H), 7.71 (d, J = 4.5 Hz, 1H), 7.56 (dd, J = 3.0, 9.3 Hz, 1H), 7.28 (d, J = 3.0 Hz, 1H), 7.24 (d, J = 7.8 Hz, 1H), 7.00-6.95 (m, 2H), 3.80 (s, 3H), 3.72 (s, 2H), 3.43 (s, 2H), 2.95-2.90 (m, 4H). Exchangeable proton from amine was not observed. NMR ^13^C (DMSO-d_6_, 75 MHz): δ ppm 165.30, 158.47, 148.03, 146.11, 145.33, 139.84, 139.35, 137.31, 131.22, 127.03, 124.40, 122.91, 122.73, 122.61, 116.96, 116.85, 116.75, 101.03, 55.48, 52.20, 48.00, 28.98, 25.67.

*BDM71369 (****9b****)* was prepared following the general procedure A using azide **5a** (28.7 mg, 0.143 mmol) and alkyne **7b** (31.1 mg, 0.143 mmol). The crude product was purified by flash-chromatography on silica gel DCM/MeOH 95:5, to give a brown oil (23.5 mg, 40%). Purity > 95%; LC-MS (EI(+)): m/z 418 [M+H]+; RT=1.98 min; NMR ^1^H (CDCl_3_, 300 MHz): δ ppm 8.88 (d, J = 4.8 Hz, 1H), 8.12 (d, J = 9.0 Hz, 1H), 7.85 (bs, 1H), 7.49-7.41 (m, 2H), 7.27 (m, 1H, under solvent peak), 6.88-6.79 (m, 3H), 4.21 (s, 4H), 3.84-3.80 (m, 5H), 3.11 (bs, 4H). Exchangeable proton from amine was not observed. NMR ^13^C (CDCl_3_, 75 MHz): δ ppm 158.35 (2C), 147.49, 146.44, 146.31, 143.61, 143.01, 139.93, 132.12, 131.59, 123.69, 123.48, 121.60, 117.40, 116.48 (2xC), 100.69, 64.42 (2xC), 55.82, 47.70, 29.80, 25.82. Traces of solvents are identified: dichloromethane (5.75 ppm) and grease (1.24 ppm and 0.84 ppm).

*BDM71346 (****9c****)* was prepared following the general procedure A using azide **5a** (45.3 mg, 0.226 mmol) and alkyne **7c** (52.1 mg, 0.226 mmol). The crude product was purified by flash-chromatography on silica gel DCM/MeOH 95:5, to give a brown oil (24.4 mg, 25%). Purity > 95%; LC-MS (EI(+)): m/z 431 [M+H]+; RT=1.82 min; NMR ^1^H (DMSO-d_6_, 300 MHz): δ ppm 10.70 (br s, 1H), 8.92 (d, J = 4.5 Hz, 1H), 8.60 (s, 1H), 8.11 (d, J = 9.3 Hz, 1H), 7.71 (d, J = 4.5 Hz, 1H), 7.56 (dd, J = 2.7, 9.3 Hz, 1H), 7.27 (d, J = 2.7 Hz, 1H), 6.95-6.87 (m, 3H), 4.53 (s, 2H), 3.81 (s, 3H), 3.72 (s, 2H), 2.96 (s, 4H). Exchangeable proton from amine was not observed. NMR ^13^C (DMSO-d_6_, 75 MHz): δ ppm 165.00, 158.48, 148.04, 145.92, 145.34, 142.16, 139.35, 134.20, 131.24, 127.03, 124.45, 122.92, 122.76, 116.87, 115.77, 115.66, 101.03, 66.77, 55.49, 51.93, 47.79, 29.00, 25.38. Traces of solvents were identified: chloroform (8.31 ppm, 79.2 ppm), water (3.33 ppm), dimethylformamide (7.95 ppm, 2.89 ppm and 2.72 ppm) and grease (1.24 ppm and 0.84 ppm).

*BDM71403 (****10a****)* was prepared following the general procedure C using azide **5b** (20.1 mg, 0.100 mmol) and alkyne **7a** (24.6 mg, 0.100 mmol). The crude product was purified by flash-chromatography on silica gel DCM/MeOH 94:6, to give a yellow solid (10.4 mg, 23%). Purity > 95%; LC-MS (EI(+)): m/z 448 [M+H]+; RT=1.90 min; NMR ^1^H (DMSO-d_6_, 300 MHz): δ ppm 10.50 (s, 1H), 9.11 (s, 1H), 8.96 (d, J = 4.8 Hz, 1H), 8.42 (d, J = 9.3 Hz, 1H), 8.22 (d, J = 4.8 Hz, 1H), 7.42 (d, J = 9.3 Hz, 1H), 7.23 (d, J = 7.8 Hz, 1H), 6.94-6.95 (m, 2H), 3.97 (s, 3H), 3.68 (brs, 2H), 3.42 (s, 2H), 2.99-2.84 (m, 4H). Exchangeable proton from amine was not observed. NMR ^13^C (DMSO-d_6_, 75 MHz): δ ppm 165.31, 162.16, 148.68, 146.02, 143.04, 140.87, 139.98, 137.80, 137.31, 132.34, 127.03, 125.27, 122.61, 117.66, 116.92, 116.73, 116.57, 54.17, 52.35, 48.20, 28.98, 25.72. Traces of water (3.33 ppm) and grease (1.24 ppm and 0.84 ppm) were identified. HRMS (Maldi-TOF): m/z calculated for [M+H]^+^ = 448.1556; found for [M+H]^+^ = 448.1566.

*BDM71404 (****10b****)* was prepared following the general procedure C using azide **5b** (20.1 mg, 0.100 mmol) and alkyne **7b** (21.73 mg, 0.100 mmol). The crude product was purified by flash-chromatography on silica gel DCM/MeOH 95:5, to give a yellow oil (9.4 mg, 22%). Purity > 95%; LC-MS (EI(+)): m/z 419 [M+H]+; RT=2.03 min; NMR ^1^H (DMSO-d_6_, 300 MHz): δ ppm 9.10 (s, 1H), 8.95 (d, J = 4.8 Hz, 1H), 8.41 (d, J = 9.3 Hz, 1H), 8.21 (d, J = 4.8 Hz, 1H), 7.42 (d, J = 9.0 Hz, 1H), 6.80 (s, 1H), 6.75 (d, J = 0.9 Hz, 2H), 4.18 (s, 4H), 3.97 (s, 3H), 3.62 (s, 2H), 2.94 (t, J = 6.3 Hz, 2H), 2.83 (t, J = 6.3 Hz, 2H). Exchangeable proton from amine was not observed. NMR ^13^C (DMSO-d_6_, 75 MHz): δ ppm 162.15, 148.67, 146.10, 143.04, 143.02, 140.86, 137.79, 133.74, 132.31, 125.26, 120.79, 117.63, 116.57 (3xC), 116.54, 64.03 (2xC), 54.17, 52.10, 47.94, 25.67. Traces of water (3.33 ppm) were identified.

*BDM71367 (****11a****)* was prepared following the general procedure B using azide **5a** (30.2 mg, 0.151 mmol) and alkyne **8a** (39.0, 0.150 mmol) to afford an off-white powder (65.1 mg, 94%). Purity > 95%; LC-MS (EI(+)): m/z 461 [M+H]+; RT=2.10 min; NMR ^1^H (DMSO-d_6_, 300 MHz): δ ppm 10.70 (brs, 1H), 8.94 (brs, 1H), 8.66 (s, 2H), 8.12 (d, J = 8.7 Hz, 1H), 7.70 (d, J = 3.3 Hz, 1H), 7.57 (d, J = 8.7 Hz, 1H), 7.48-7.38 (m, 3H), 7.28 (s, 1H), 3.79 (s, 3H), 3.70-3.61 (m, 2H), 3.50 (s, 2H), 3.11-3.02 (m, 2H). NMR ^13^C (DMSO-d_6_, 75 MHz): δ ppm 165.60, 165.01, 158.52, 148.06, 145.37, 139.78, 139.36, 137.39, 133.27, 131.23, 126.95, 124.58, 122.98, 122.82, 120.95, 117.01, 116.50, 116.36, 100.95, 55.49, 28.62, 25.48 (2xC). Traces of water (3.33 ppm) were identified.

*BDM71365 (****11b****)* was prepared following the general procedure B using azide **5a** (30.0 mg, 0.150 mmol) and alkyne **8b** (34.9, 0.151 mmol) to afford an off-white powder (62.6 mg, 96%). Purity > 95%; LC-MS (EI(+)): m/z 432 [M+H]+; RT=2.22 min; NMR ^1^H (DMSO-d_6_, 300 MHz): δ ppm 8.92-8.10 (m, 4H), 7.69-7.25 (m, 4H), 6.89 (s, 1H), 4.26 (s, 4H), 3.78-3.63 (m, 4H), 3.35-3.05 (m, 4H). NMR ^13^C (DMSO-d_6_, 75 MHz): δ ppm 165.51, 158.51 (2xC), 148.01, 145.93, 145.46, 145.35, 142.87, 139.39, 131.23, 127.56, 124.58, 123.01, 122.86, 120.62, 117.02, 116.71, 116.20, 100.92, 64.32 (2xC), 64.00, 55.48, 25.58.

##### Solubility

10μL of a 10 mM solution in DMSO of the compound are diluted in 490 μL of PBS pH 7.4 in Matrix tube (in triplicate) or organic solvent (ACN, MeOH) in Matrix tube (in triplicate). The tubes are gently shaken 24h at room temperature. Then the tubes are centrifuged for 5 minutes at 4000 rpm and filtered over 0.45 μm filters (Millex-LH Millipore). 20 μL of each tube are diluted in 180 μL of MeOH. Analysis is performed thanks to a UPLC-MS/MS system (Waters) under SIM detection using the parameters optimized for each compound. HPLC analysis was performed using an Aquity C18 (50x2.1 mm, 1.8 μm); the gradient and the mobile phase (flow rate 600 μL/min^-1^) used are determined in order to detect the compound of interest with satisfying retention time and peak shape. Acquisition and analysis of data were performed with Masslynx software. The solubility is determined by the ratio of mass signal area PBS/ organic solvent.

##### LogD evaluation

40 μL of a 10 mM solution in DMSO of the compound were diluted in 1.960 mL of a 1/1 octanol/PBS at pH 7.4 solution. The mixture was gently shaken 2h at room temperature. 20 μL of each solution was diluted in 480 μμL of MeOH and analyzed by LC-MS. Each compound is tested in triplicate. Analysis is performed thanks to a UPLC-MS/MS triple-quadrupole system (Waters) under SIM or MRM detection using the parameters optimized for each compound. HPLC analysis was performed using an Aquity C18 (50x2.1 mm, 1.8 μm); the gradient and the mobile phase (flow rate 600μL/min^-1^) used are determined in order to detect the compound of interest with satisfying retention time and peak shape. Acquisition and analysis of data were performed with MS Workstation™ software (version 6.3.0 or higher). LogD was determined as the logarithm of the ratio of concentrations of product in octanol and PBS, determined by mass signals.

### Quantification and statistical analysis

Each data point represents three trials, usually with the same stocks of enzymes and substrates. Error bar represent the standard error of the mean (SEM).

### Additional resources

• The BDM71403-bound and gepotidacin-bound *Mtb* gyrase coordinates have been submitted to the Protein Data Bank (https://www.rcsb.org/) with PDB IDs 8S70 and 8S7K, respectively.
• Corresponding EM maps have been submitted to Electron Microscopy Data Bank (https://www.ebi.ac.uk/pdbe/emdb/) with IDs EMD-19782 and EMD-19777, respectively.
• Other data are available from corresponding authors upon request.
