# Supplementary material for: Molecular mechanism of a triazole-containing inhibitor of Mycobacterium tuberculosis DNA gyrase
Source: iScience. 2024 Sep 16;27(10):110967. doi: 10.1016/j.isci.2024.110967 (PMC11489056; doi:10.1016/j.isci.2024.110967)
Supplement: Document S1. Figures S1–S9, Tables S1–S3, and Data S1 [file mmc1.pdf]

iScience, Volume *27*

Supplemental information

Molecular mechanism of a triazole-containing inhibitor of *Mycobacterium tuberculosis* DNA gyrase

Antoine Gedeon, Emilie Yab, Aurelia Dinut, Elodie Sadowski, Estelle Capton, Aurore Dreneau, Julienne Petit, Bruna Gioia, Catherine Piveteau, Kamel Djaout, Estelle Lecat, Anne Marie Wehenkel, Francesca Gubellini, Ariel Mechaly, Pedro M. Alzari, Benoît Deprez, Alain Baulard, Alexandra Aubry, Nicolas Willand, and Stéphanie Petrella

Table S1. MICs of compound BDM71403 and ciprofloxacin against ESKAPE strains

ATCC or CFPL number	Strain	Resistance profile	BDM71403 (13a) MIC (µg/mL)	Ciprofloxacin MIC (µg/mL)	Ciprofloxacin MIC/BDM71403 MIC (µg/mL)
ATCC 25922	*E. coli*	Wild-type	1	<0.06	<0.06
8137	*E. coli*	Wild-type	1	<0.06	<0.06
8138	*E. coli*	Penicillin resistant	1	<0.06	<0.06
8141	*E. coli*	Wild-type	1	<0.06	<0.06
8157	*E. coli*	Penicillin and fluoroquinolone resistant	0.5	16	32
9003	*E. coli*	BLSE	1	16	16
10273	*E. coli*	KPC-2	1	>32	>32
10269	*E. coli*	NDM-1	1	32	32
10385	*E. coli*	NDM-1	<0.06	32	>533
10386	*E. coli*	NDM-1	1	>32	>32
10270	*K. pneumoniae*	VIM	4	>32	>8
10272	*K. pneumoniae*	0XA-48	2	0.5	0.25
10277	*K. pneumoniae*	KPC-2	2	32	16
8127	*P. aeruginosa*	Fluoroquinolone resistant	4	8	2
8131	*P. aeruginosa*	Wild-type	4	0.25	0.06
8132	*P. aeruginosa*	Multi-resistant with protein-D2 porin defect	2	32	16
8133	*P. aeruginosa*	Wild-type	8	0.25	0.03
8134	*P. aeruginosa*	Fluoroquinolone resistant	2	32	16
8135	*P. aeruginosa*	Wild-type	16	0.5	0.03
8136	*P. aeruginosa*	Multi-resistant with protein-D2 porin defect	4	32	8
09010	*A. baumannii*	VEB-1	1	>32	>32
09011	*A. baumannii*	Multi-resistant	N.D.	32	-
10275	*A. baumannii*	VIM-4	<0.06	N.D.	-
8143	*S. aureus*	Methicillin, kanamycin and tobramycin resistant	0.125	1	8
8146	*S. aureus*	Methicillin, kanamycin resistant	0.25	1	4
8147	*S. aureus*	Wild-type	4	16	4
8148	*S. aureus*	Methicillin and fluoroquinolones resistant	0.25	>32	>128
8149	*S. aureus*	Wild-type	0.25	0.5	2
8237	*S. aureus*	Wild-type	0.25	0.5	2
8238	*S. aureus*	Wild-type	0.25	0.5	2
8239	*S. aureus*	Methicillin resistant	0.5	>32	>64

8240	*S. aureus*	Methicillin and fluoroquinolones resistant	0.25	>32	>128
8241	*S. aureus*	Methicillin, kanamycin, tobramycin and fluoroquinolones resistant	0,5	>32	>64
09001	*E. faecium*	Multi-resistant	<0.06	>32	>533
09002	*E. faecium*	Multi-resistant	0.125	>32	>256

Table S2. Summary of data collection and refinement parameters

Dataset	BDM71403-bound complex	Gepotidacin-bound complex
PDB	8S70	8S7K
EMDB	EMD-19782	EMD-19777
**Data collection and processing**		
Microscope	TITAN KRIOS2 EMBL	TITAN KRIOS
Voltage (kV)	300	300
Camera	Quantum-K3 camera (Gatan)	Quantum-K3 camera (Gatan)
Magnification	130,000	105,000
Nominal defocus range (µm)	-0.9 to -1.9 (0.1 step)	-0.8 to -2.8 (0.2 step)
Electron exposure (e–/Å^2)	49	40
Number of frames collected (no.)	40	40
Pixel size (Å)	0.645	0.860
Micrographs (no.)	12,888	7,900
Total particle images (no.)	987,000	830,271
**Refinement**		
Particles per class (no.)	461,305	319,346
Map resolution (Å), 0.143 FSC	2.8	3.2
**Model composition**		
Non-hydrogen atoms	11,253	11,314
Protein residues	1,347	1,341
Nucleotide residues	34	36

Ligands	BDM:1	JHN: 1<br>MG: 2
***B* factors (Å^2)**		
Protein	96.5	139.28
Nucleotide	59.7	77.26
Ligand	108.8	66.65
**R.m.s deviations**		
Bond lengths (Å)	0.004	0.003
Bond angles (°)	0.595	0.549
**Validation**		
MolProbity score	2.26	1.64
All-atom clashscore	12.95	5.5
**Ramachandran plot**		
Favored (%)	97.23	95.02
Allowed (%)	2.77	4.98
Disallowed (%)	0	0

Table S3. Asymmetric oligonucleotides sequences used for cryo-EM complexes. Only the 5’-3’ strand is displayed.

ME-73b	TTCGGCGAGAAGCAGGCCATTATCGCCGGCATGGCGGCCGACGCGCTGGGCTACGTCTTGCTGGCGTTCGCGA
ME-77b	CGCGAGGCTGGATGGCCTTCCCCATTATGATTCTTCTCGCTTCCGGCGGCATCGGGATGCCCGCGTTGCAGGCCATG

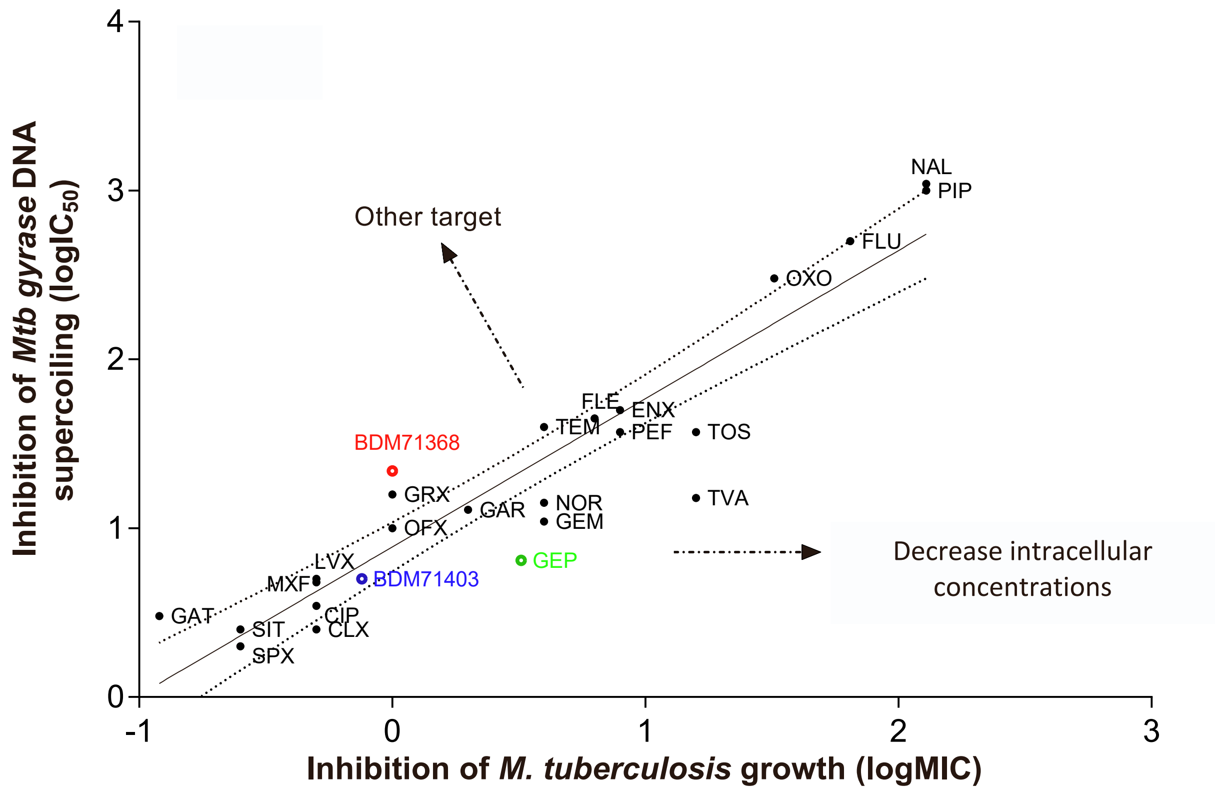

Figure S1. Correlation curve between *in vitro* activity (IC_{50} for DNA supercoiling for wild-type *Mtb* gyrase) versus MICs (for wild-type *Mtb* H37Rv) for FQ and NBTI. Dotted lines represent the confidence interval for 95% of the regression. CIP, ciprofloxacin; CLX, clinafloxacin; ENX, enoxacin; FLE, fleroxacin; FLU, flumequine; GAR, garenoxacin; GAT, gatifloxacin; GEM, gemifloxacin, GEP, gepotidacin; GRX, grepafloxacin; LVX, levofloxacin; MXF, moxifloxacin; NAL, nalidixic acid; NOR, norfloxacin, OFX, ofloxacin; OXO, oxolinic acid; PEF, pefloxacin; PIP, pipemidic acid; SIT, sitafloxacin; SPX, sparfloxacin; TEM, temafloxacin; TOS, tosufloxacin; TVA, trovafloxacin

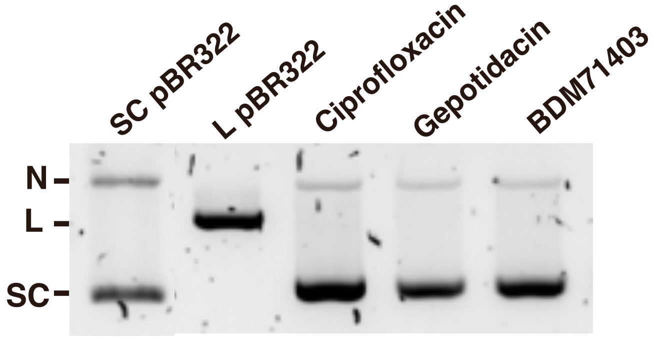

Figure S2. pBR322 coiling state in the sole presence of ciprofloxacin, gepotidacin or BDM71403 in the absence of DNA gyrase. 250 ng of supercoiled DNA was incubated in the presence of 25 µM of the different molecules for one-hour et 37°C, then treated as the same protocol as DNA cleavage assays (incubation with 0.01 mg/mL proteinase K and 0.2% SDS for one hour at 37°C) and migrated on 1% agarose gel in the presence 0.5 µg/mL of SYBR™ Safe. Supercoiled (SC) and linearized (L) pBR322 were also migrated as controls at 250 ng.

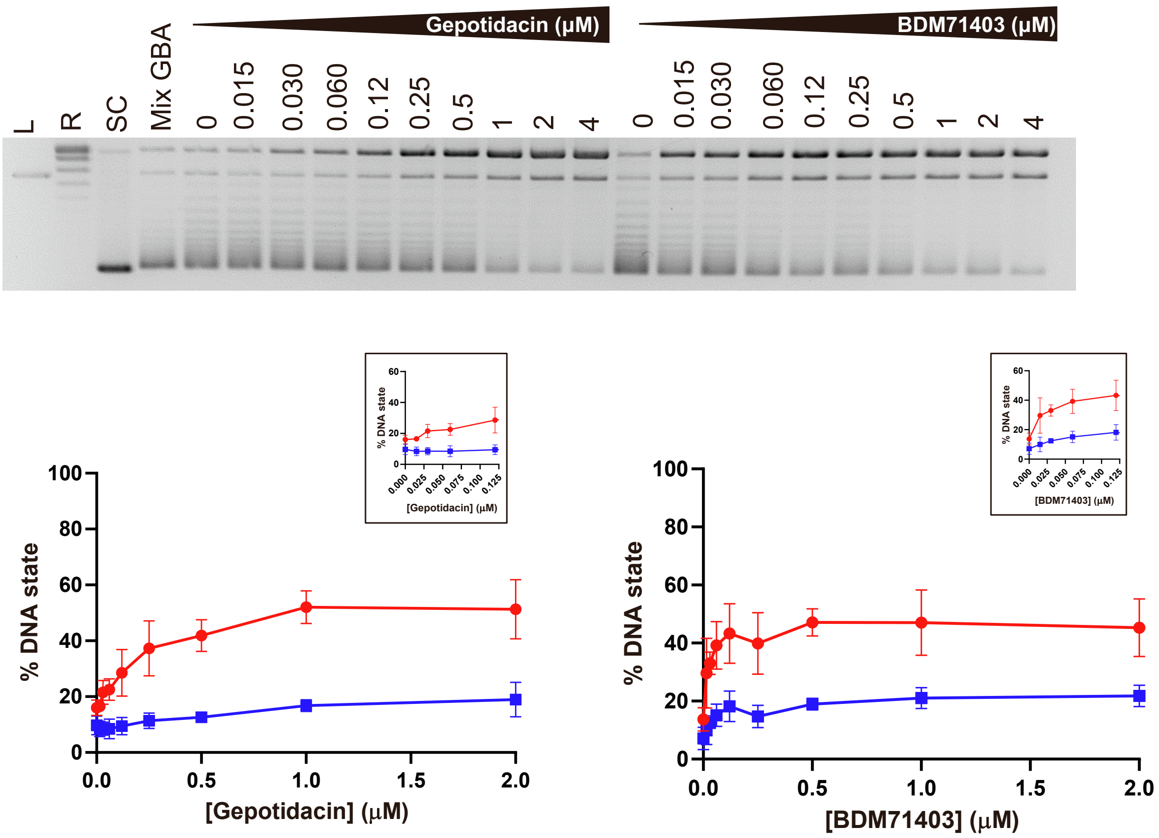

Figure S3. DNA cleavage assay on pBR322 with *Mtb* gyrase in the presence of variable concentrations of gepotidacin or BDM71403. N, nicked DNA; L, linear DNA; SC, supercoiled DNA. Error bars represent standard deviations from three independent experiments (*n*=3). Percentages of single-strand breaks (SSB) are represented in red AND double-strand breaks (DSB) in blue. Note that the migration for this gel was done in the absence of intercalating agent, and DNA bands were revealed after incubation in SYBR^{TM} Safe.

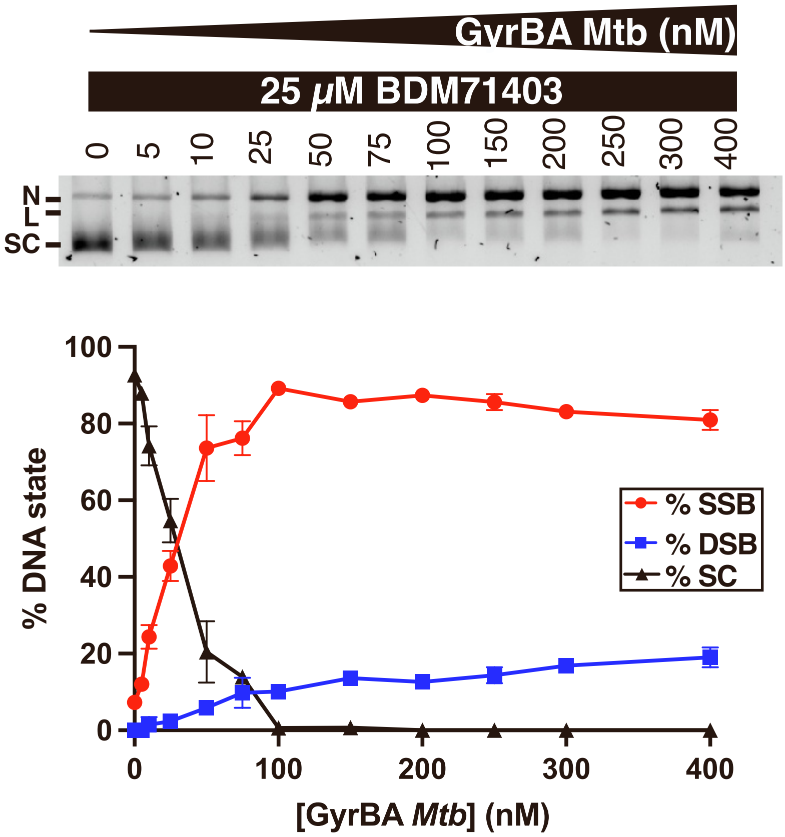

Figure S4. DNA cleavage assay on 5 nM pBR322 with variable concentration of *Mtb* gyrase in the presence of 25 µM of BDM71403. N, nicked DNA; L, linear DNA; SC, supercoiled DNA. Error bars represent standard deviations from two independent experiments (*n*=2). Percentages of single-strand breaks (SSB) are represented in red and double-strand breaks (DSB) in blue.

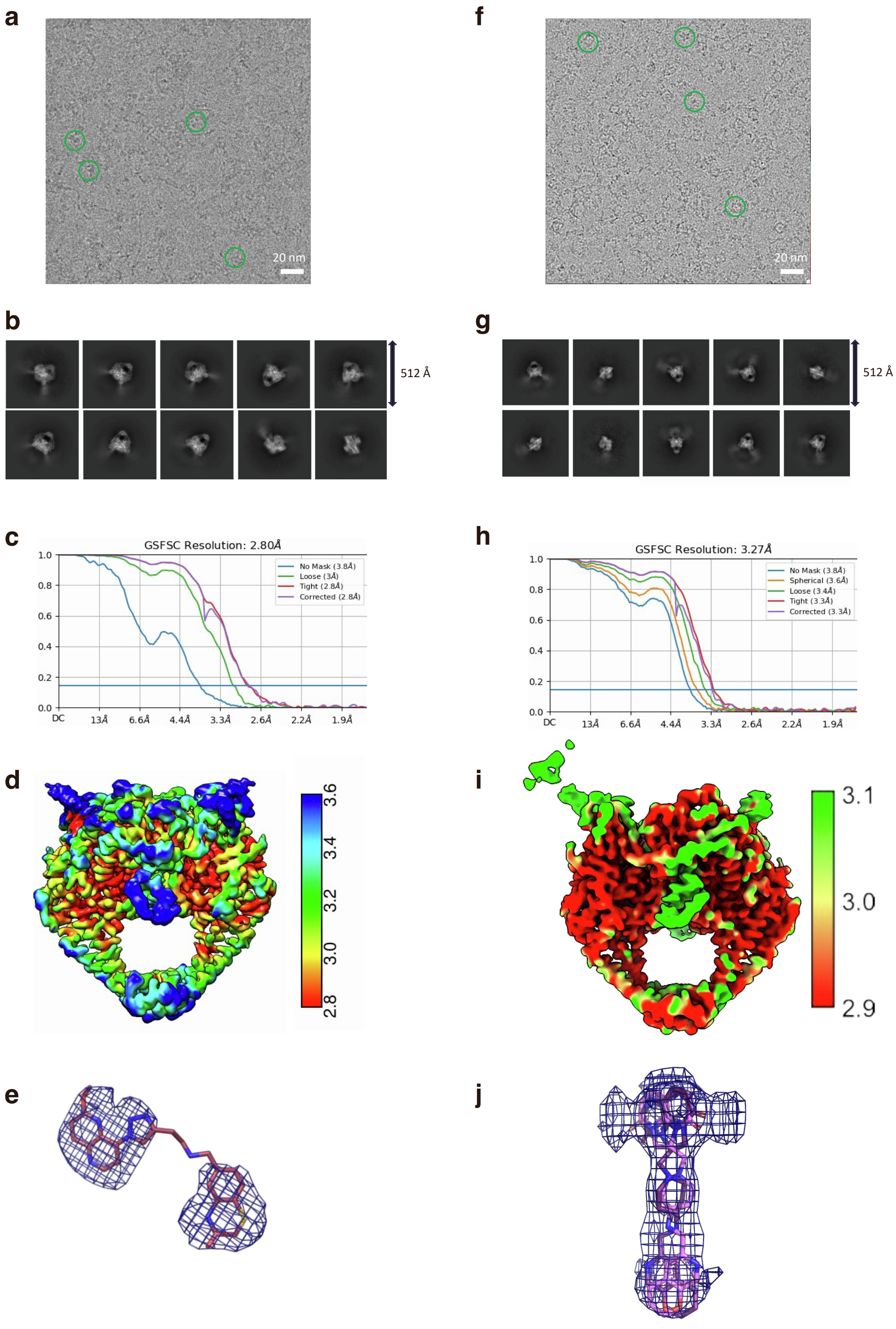

Figure S5. CryoEM data for gyrase-DNA-BDM71403 (**a-e**) and gyrase-DNA-gepotidacin (**f-j**) complexes. **a,f,** Representative micrograph with example gyrase particles indicated. **b,g**, A selection of 2D classes, box size in angstroms indicated. **c,h**, Fourier shell correlation (FSC) curves for corresponding masked or unmasked maps. **d,i**, Local resolution map contoured at level 5σ to illustrate resolution distribution from >2.8 Å next to the DNA and compounds to around 3.6 or 3.1 Å at the level of the more flexible part of the TOPRIM. **e,j**, Electron density of molecules BDM71403 (**e**) and gepotidacin (**j**) shown in blue mesh. Note that two different orientations for gepotidacin are seen.

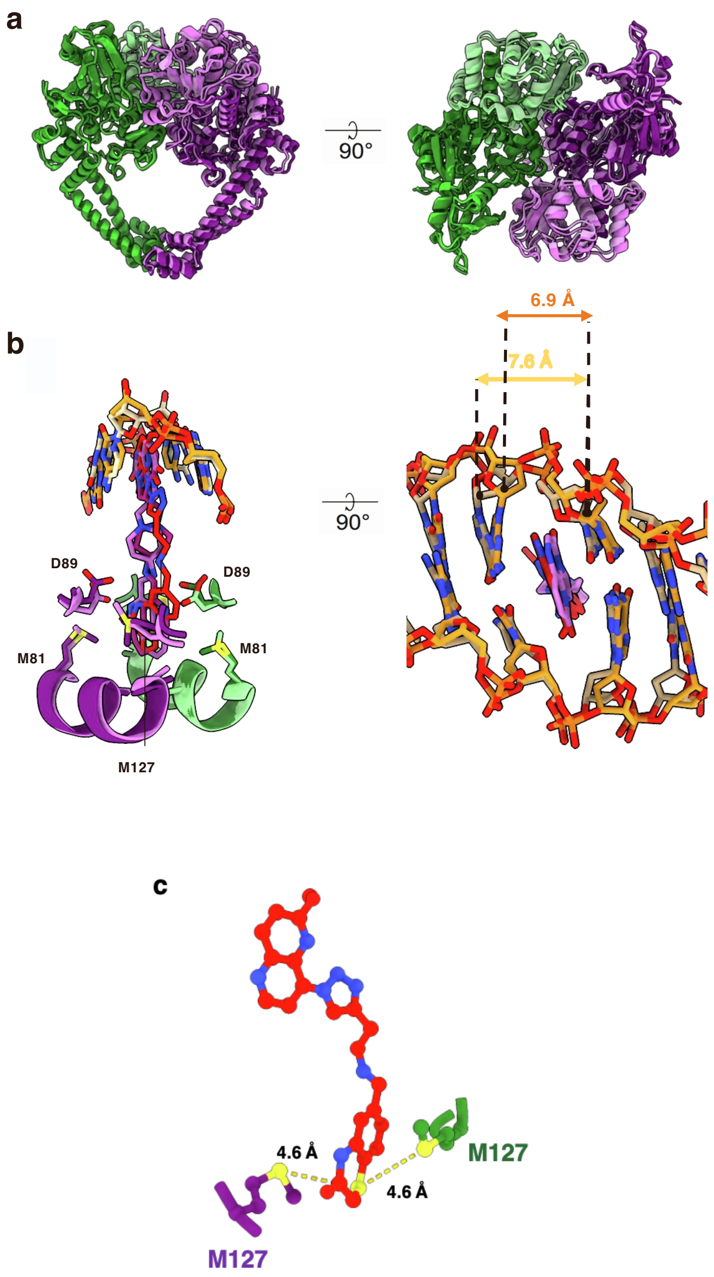

Figure S6. Superposition of *Mtb* gyrase cleavage core structures in complex with DNA-BDM71403 or DNA-gepotidacin. **a**, Superimposition of the two empty cleavage cores in cartoon representation. BRD are in green and TOPRIM domains in pink colors. **b,** Zoom on the binding pocket of BDM71403/gepotidacin. DNA from BDM71403 complex is in yellow and DNA from gepotidacin complex is in fire orange. The residues implicated in the stabilization of the drugs are in the same colors as their BRD domains. **c**, Zoom on the sulfur-sulfur interactions between the two methionine and the sulfur atom of the RHS substituent of BDM71403 (in red). The two methionine are in stick and ball representations in the same colors as their corresponding BRD domains.

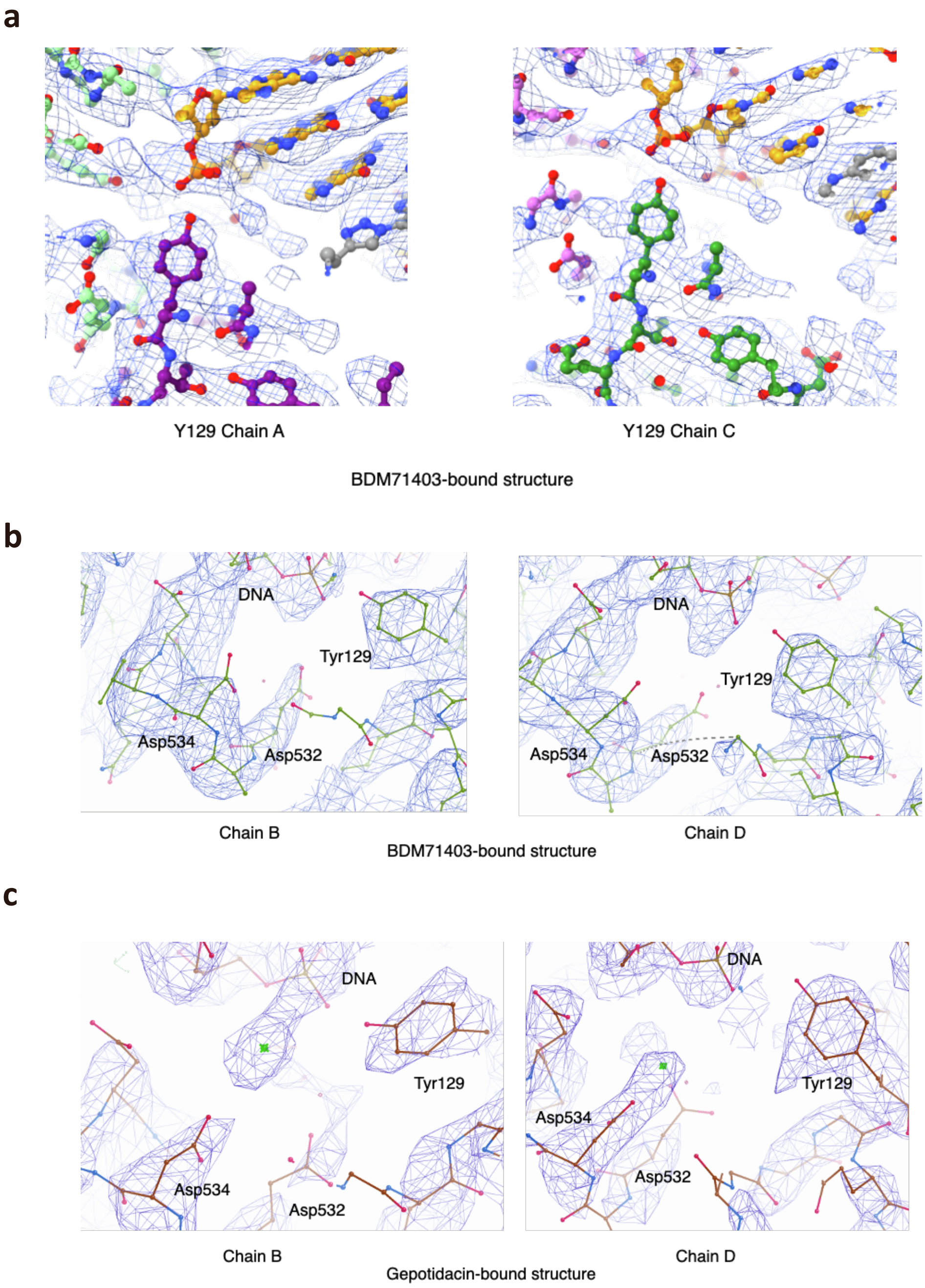

Figure S7. a, Density of the catalytic tyrosine (Y129) for the cleavage-complex structure solved at 2.8 Å resolution for BDM71403. **b** and **c**, Metal-binding site associated with cleavage of DNA. EM density of the cleavage-complex structure for BDM71403 (panel **b**) and gepotidacin (panel **c**) complexes, respectively. These maps show no density at the site of a Mg^{2+} ion in the BDM71403-bound structure, whereas in the gepotidacin-bound structure an ion can be placed. The position of this ion is not exactly the same in the two chains of the gepotidacin-bound structure. The ion is a green sphere visible in panel **c**.

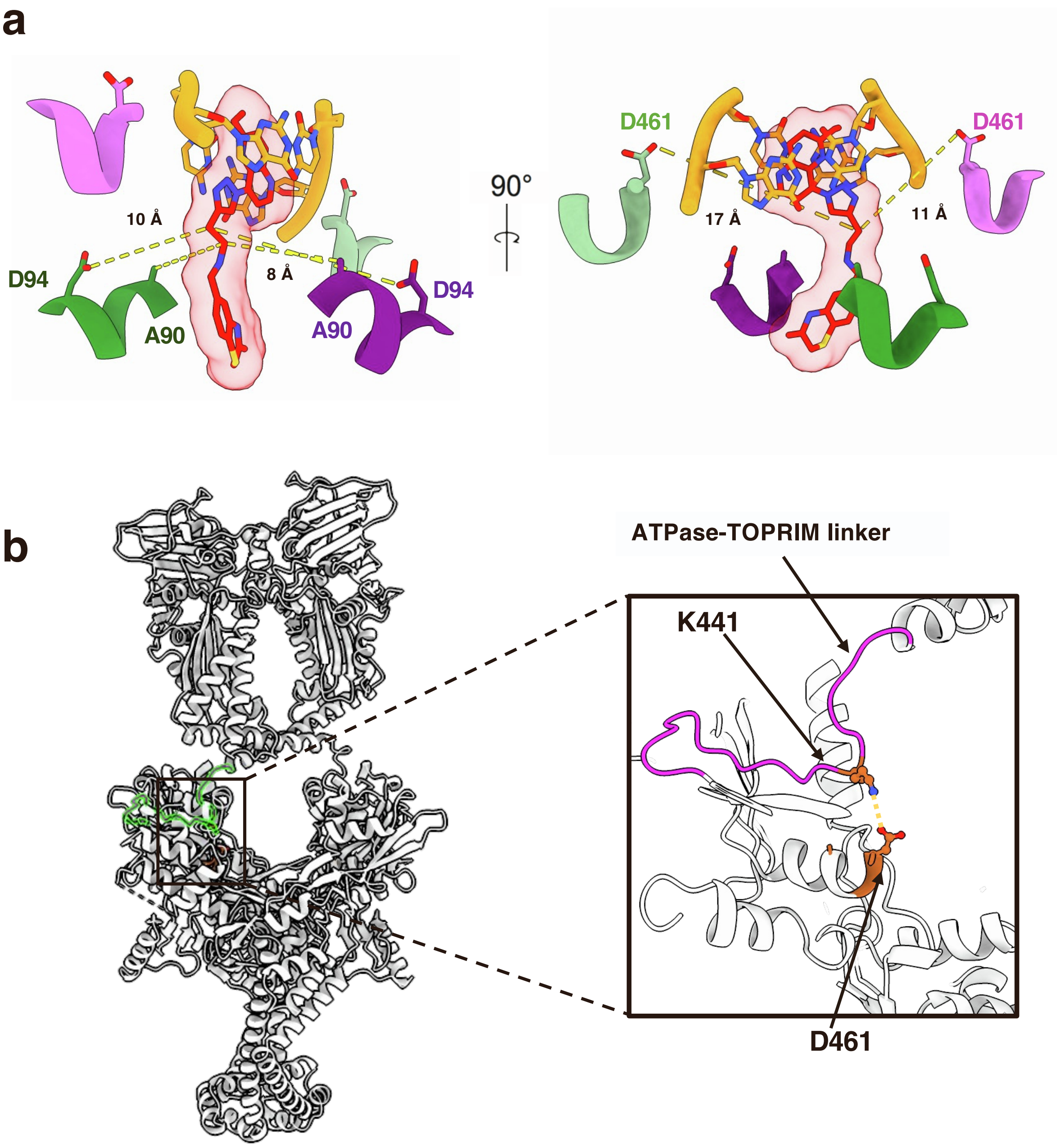

Figure S8. Zoom on the residues implicated in FQ resistance. **a,** BDM71403 binding site with DNA and residues in sticks representation and position of the residues implicated in FQ resistance, A90 and D94 from GyrA and D461 from GyrB. **b,** Full length-*Mtb* gyrase (unpublished data) with emphasis on D461 and K441 in stick representation. These two residues are implicated in a salt bridge.

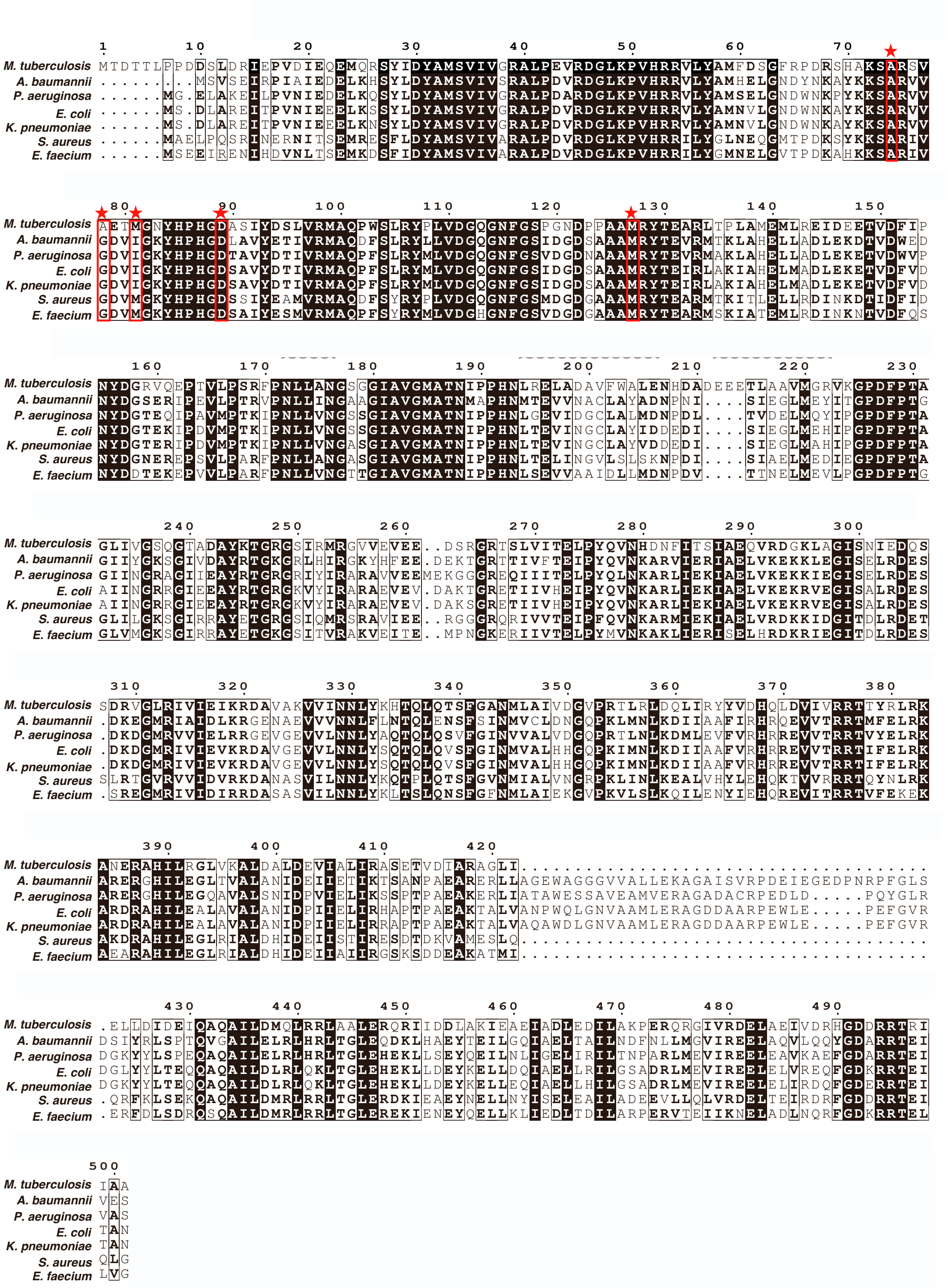

Figure S9. Conservation of BRD domain of GyrA subunits of interest in this work. **a**, Sequence alignment of *M. tuberculosis*, *A. baumannii*, *P. aeruginosa*, *E. coli*, *K. pneumoniae*, *S. aureus* and *E. faecium* GyrA subunits. UniProt accession numbers are P9WG47, X2L5Z9, P48372, P01ES4, J2X497, P20831 and A0A8B2RLX6 respectively. Numbering above sequences correspond to *Mtb* gyrase sequence. Residues implicated in NBTI binding are designated with a red star (★). Alignments generated with Clustal W 2.0[1] and ESPript 3.0[2].

Data S1: **NMR spectra for each compound, related to STAR Methods.**

^{1}H, ^{13}C NMR Spectra

BDM71368 (**9a**)

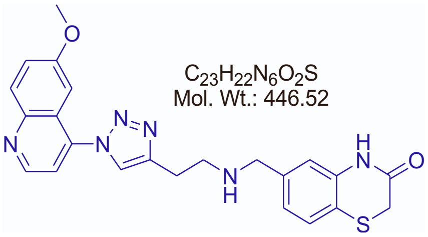

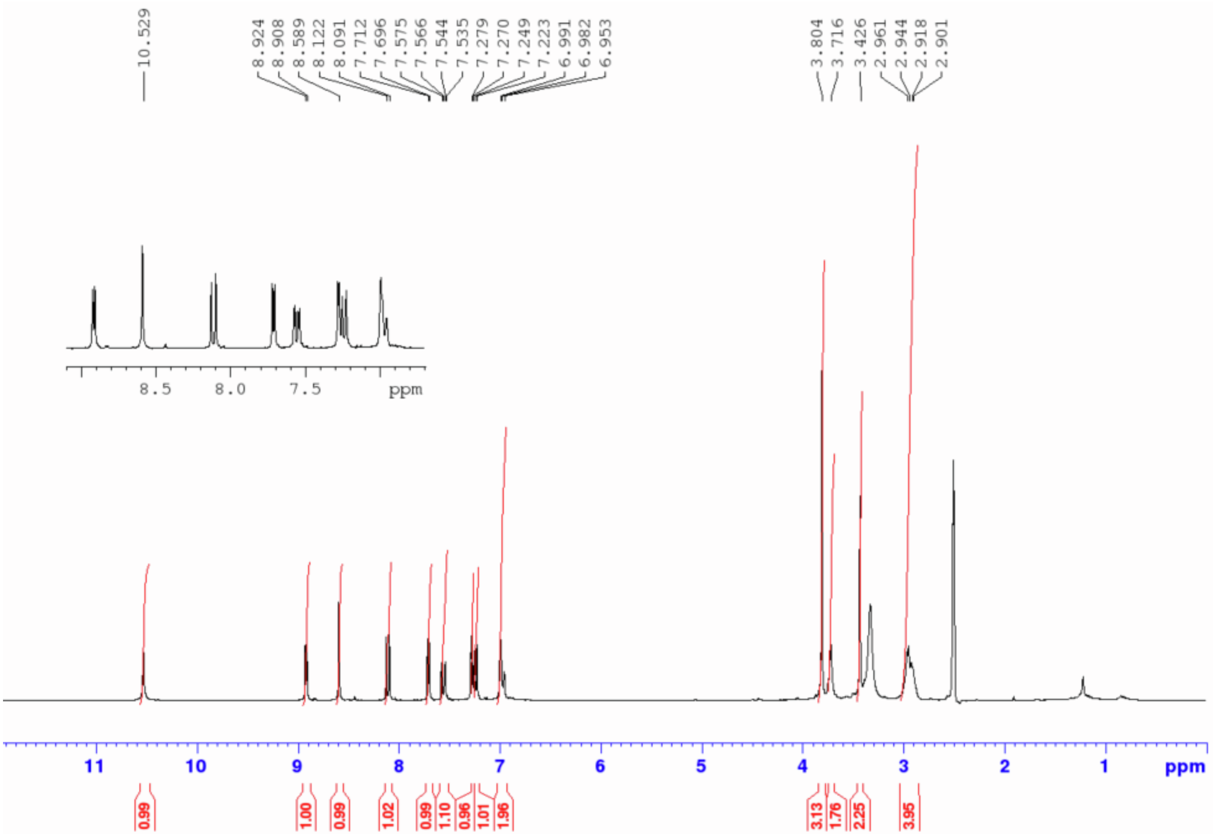

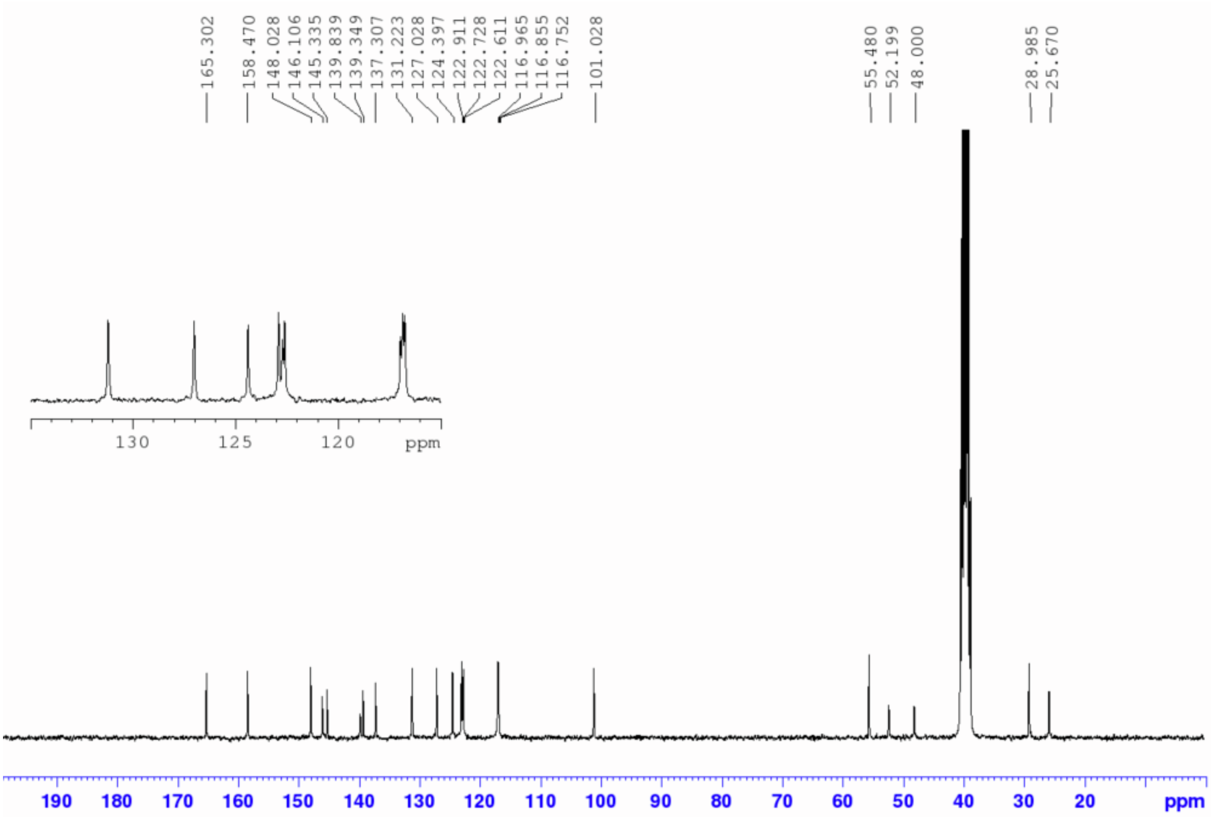
165.302
158.470
148.028
146.106
145.335
139.839
139.349
137.307
131.223
127.028
124.397
122.911
122.728
122.611
116.965
116.855
116.752
101.028
55.480
52.199
48.000
28.985
25.670
130 125 120 ppm
190 180 170 160 150 140 130 120 110 100 90 80 70 60 50 40 30 20 ppm

BDM71369 (**9b**)

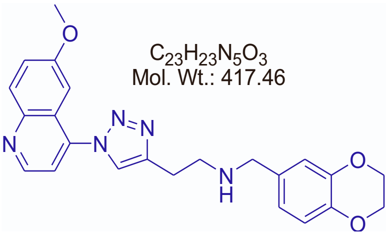

$C_{23}H_{23}N_5O_3$
Mol. Wt.: 417.46

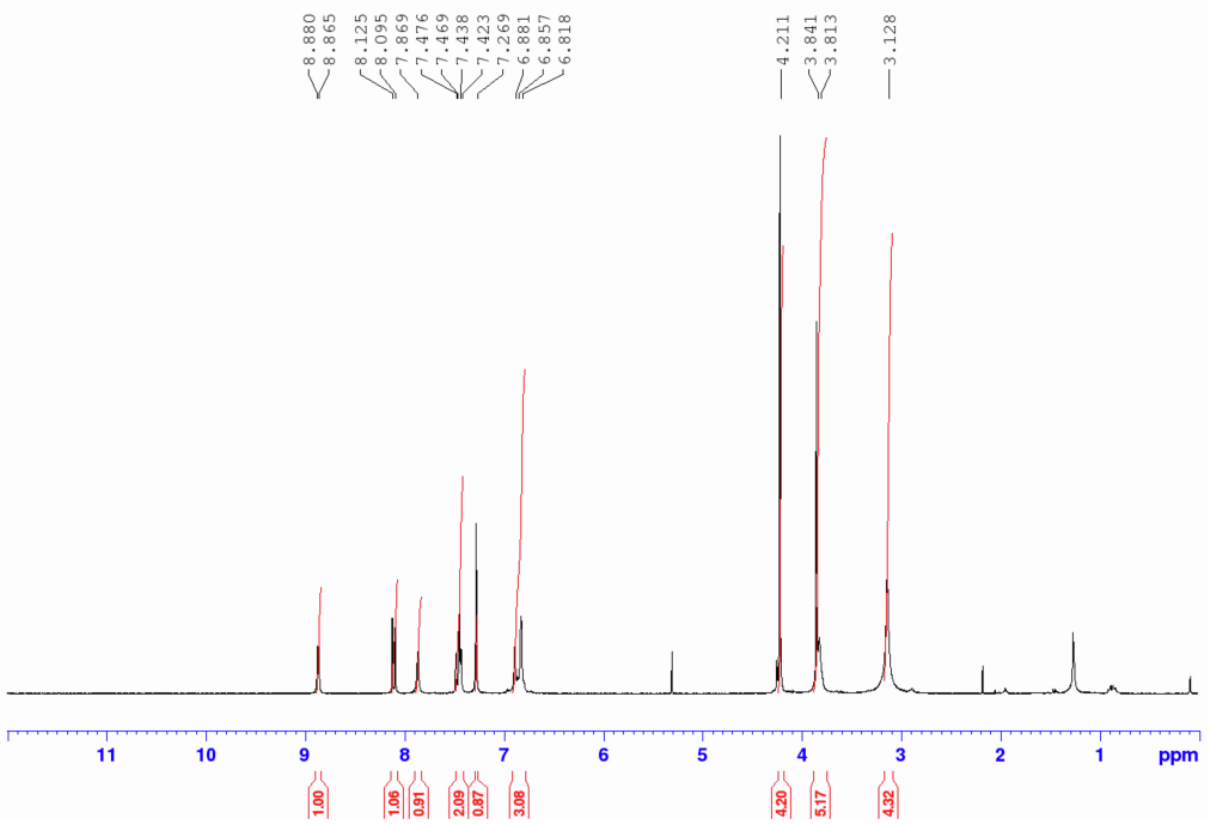

8.880
8.865
8.125
8.095
7.869
7.476
7.469
7.438
7.423
7.269
6.881
6.857
6.818
4.211
3.841
3.813
3.128
11
10
9
8
7
6
5
4
3
2
1
ppm
1.00
1.06
0.91
2.09
0.87
3.08
4.20
5.17
4.32

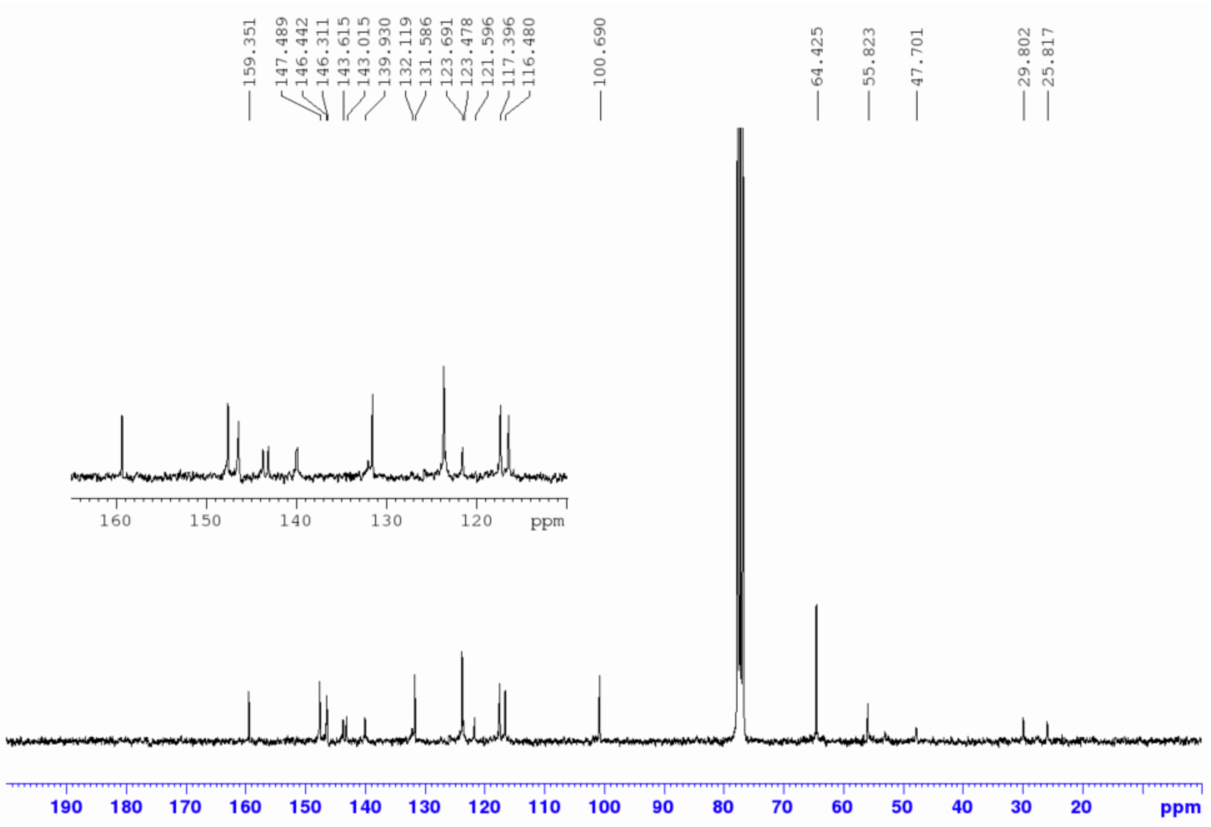
159.351
147.489
146.442
146.311
143.615
143.015
139.930
132.119
131.586
123.691
123.478
121.596
117.396
116.480
100.690
64.425
55.823
47.701
29.802
25.817
160 150 140 130 120 ppm
190 180 170 160 150 140 130 120 110 100 90 80 70 60 50 40 30 20 ppm

BDM71346 (**9c**)

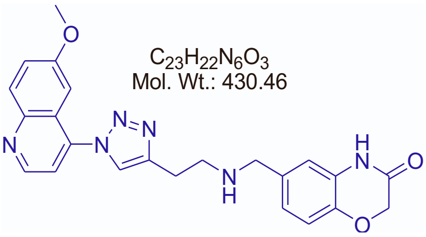

$C_{23}H_{22}N_6O_3$
Mol. Wt.: 430.46

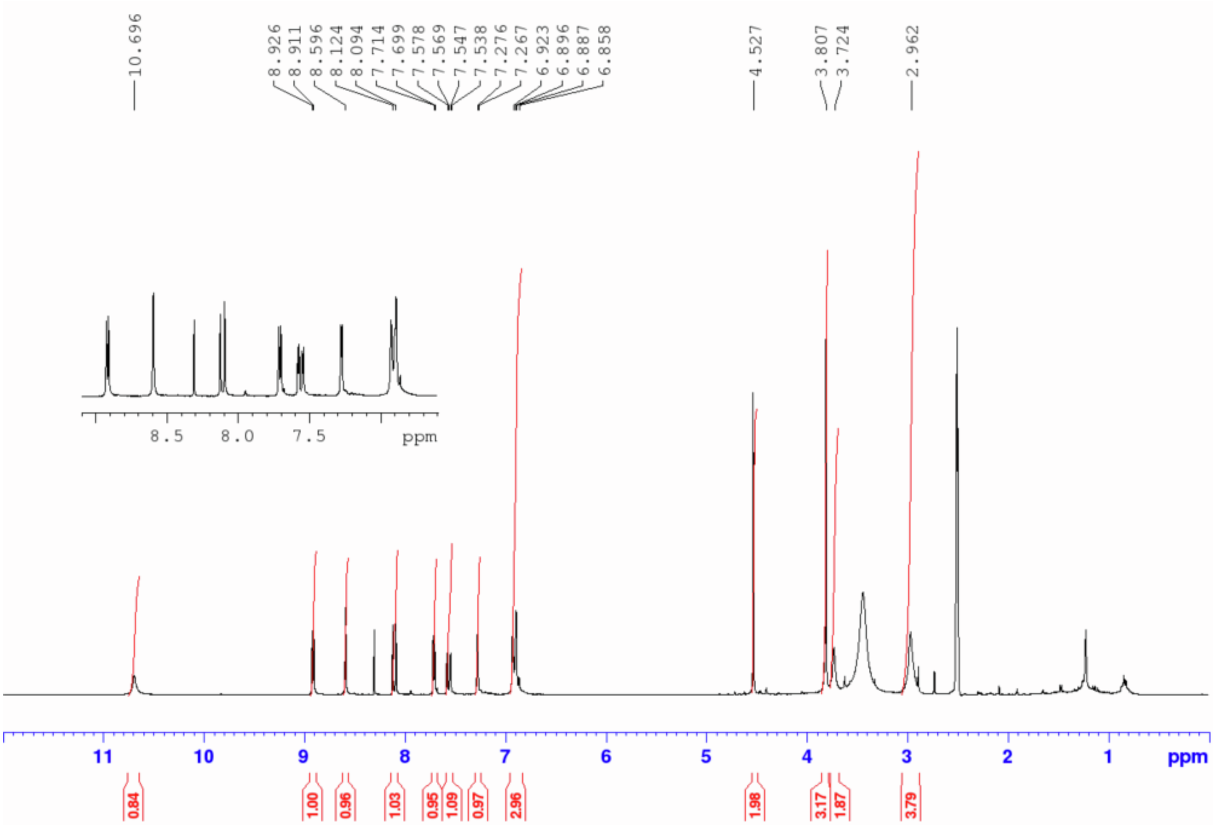

10.696
8.926
8.911
8.596
8.124
8.094
7.714
7.699
7.578
7.569
7.547
7.538
7.276
7.267
6.923
6.896
6.887
6.858
4.527
3.807
3.724
2.962
8.5
8.0
7.5
ppm
11
10
9
8
7
6
5
4
3
2
1
ppm
0.84
1.00
0.96
1.03
0.95
1.09
0.97
2.96
1.98
3.17
1.87
3.79

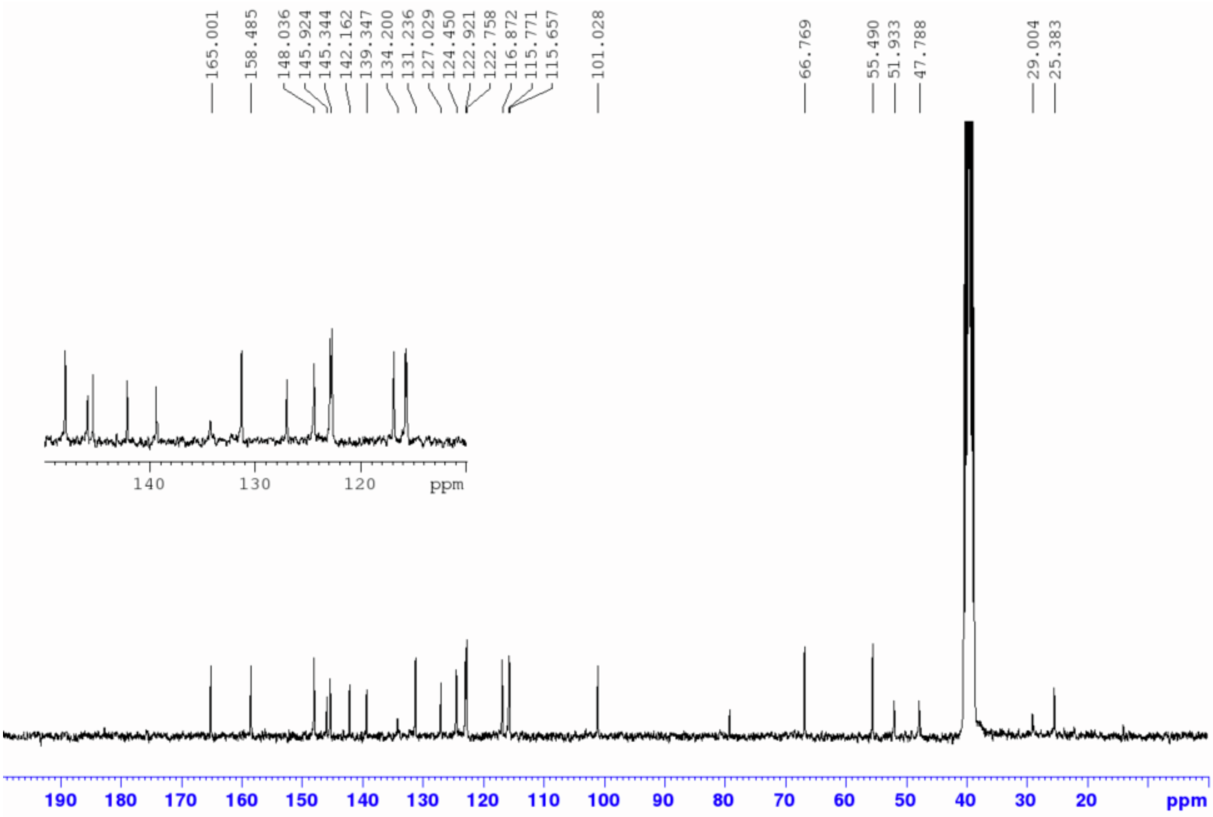
165.001
158.485
148.036
145.924
145.344
142.162
139.347
134.200
131.236
127.029
124.450
122.921
122.758
116.872
115.771
115.657
101.028
66.769
55.490
51.933
47.788
29.004
25.383
140
130
120
ppm
190
180
170
160
150
140
130
120
110
100
90
80
70
60
50
40
30
20
ppm

BDM71403 (**10a**)

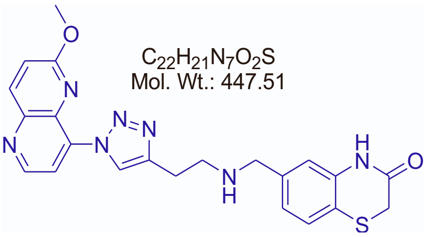

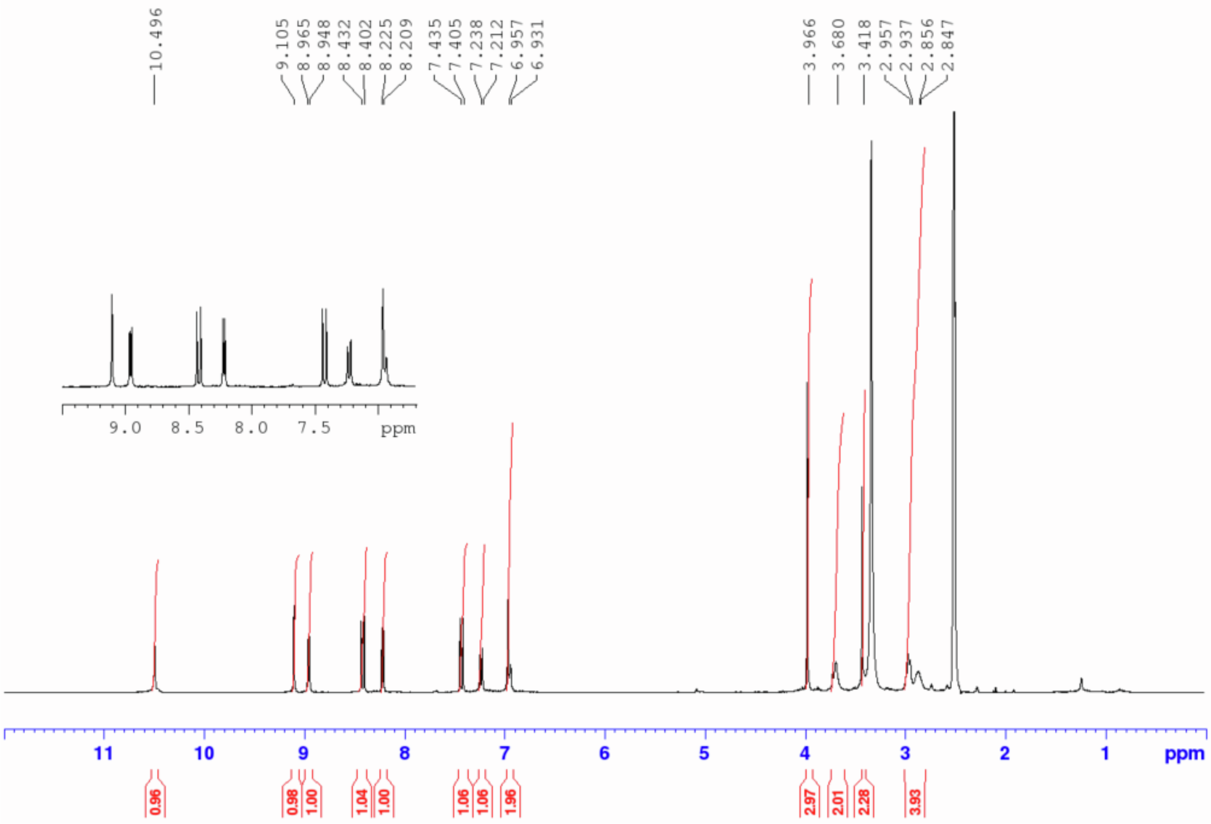

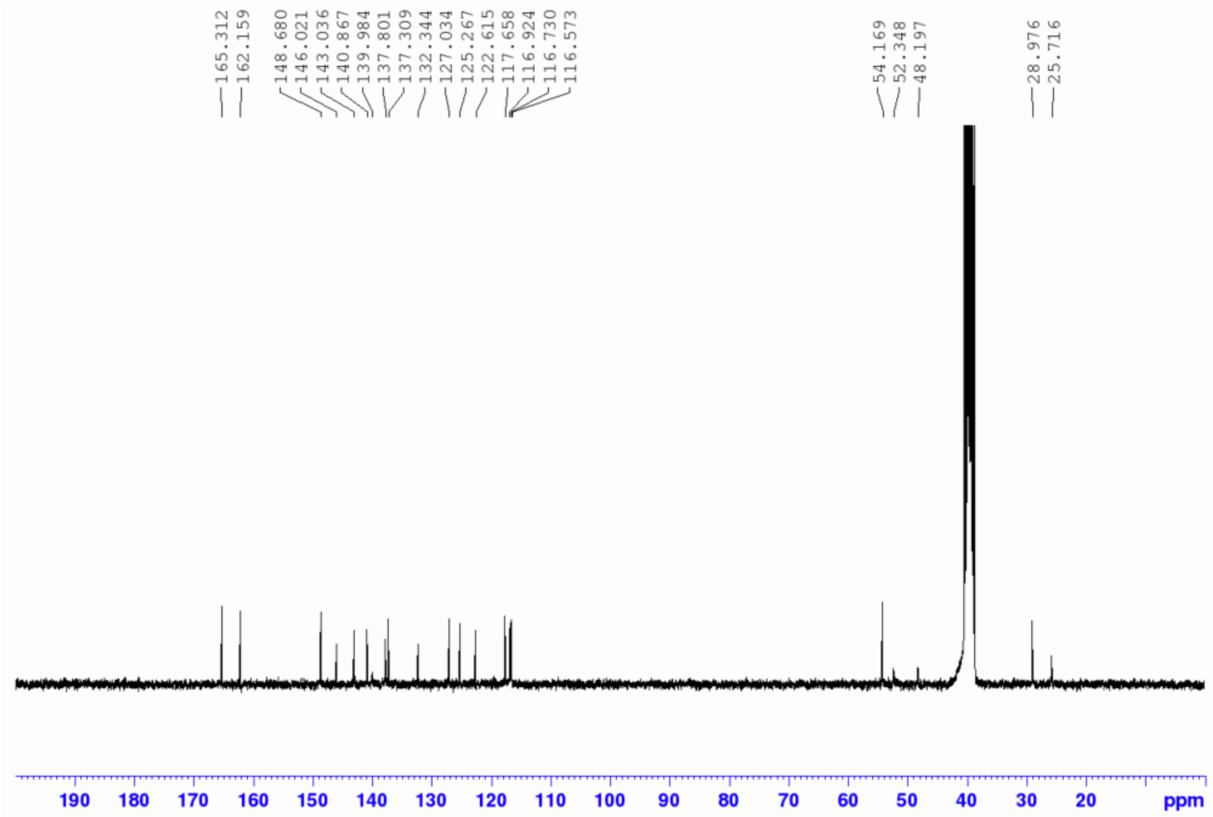

165.312
162.159
148.680
146.021
143.036
140.867
139.984
137.801
137.309
132.344
127.034
125.267
122.615
117.658
116.924
116.730
116.573
54.169
52.348
48.197
28.976
25.716
190 180 170 160 150 140 130 120 110 100 90 80 70 60 50 40 30 20 ppm

BDM71404 (**10b**)

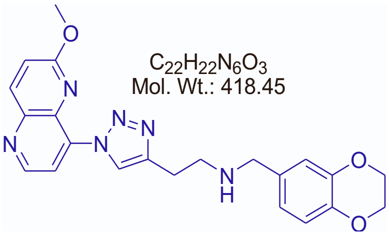

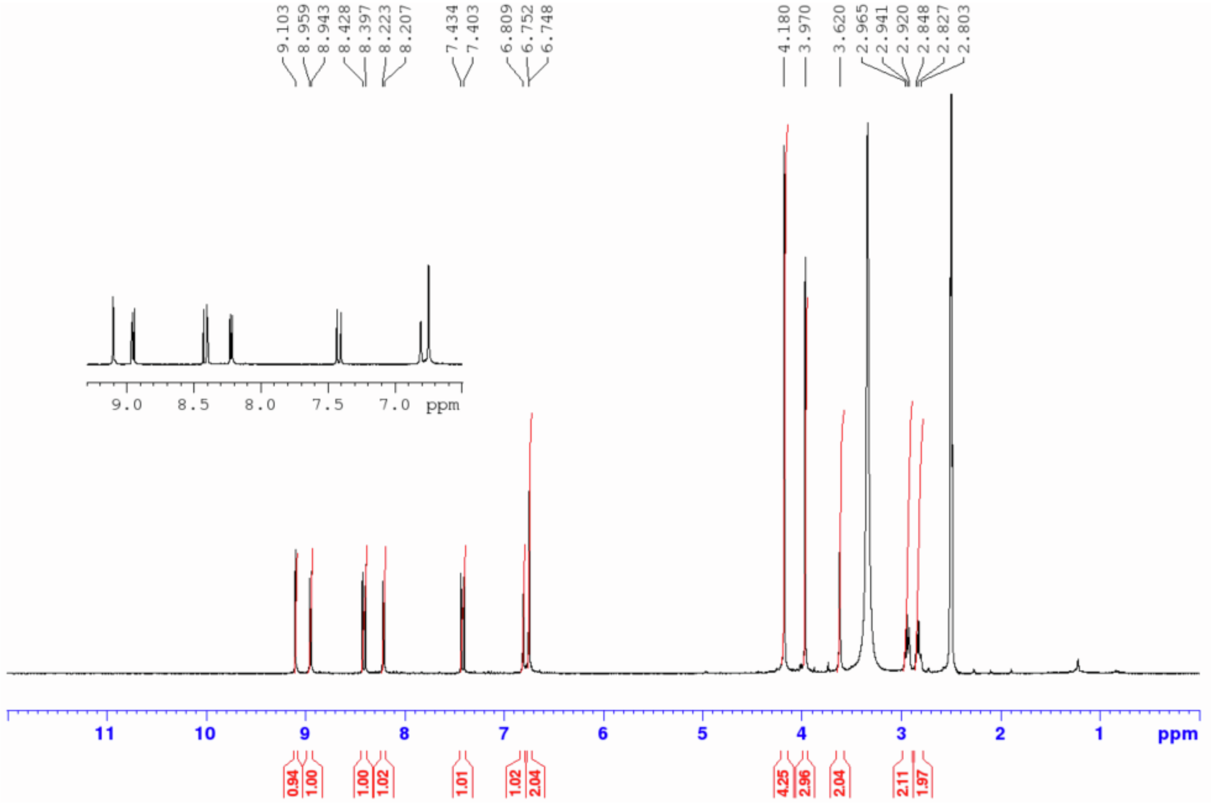

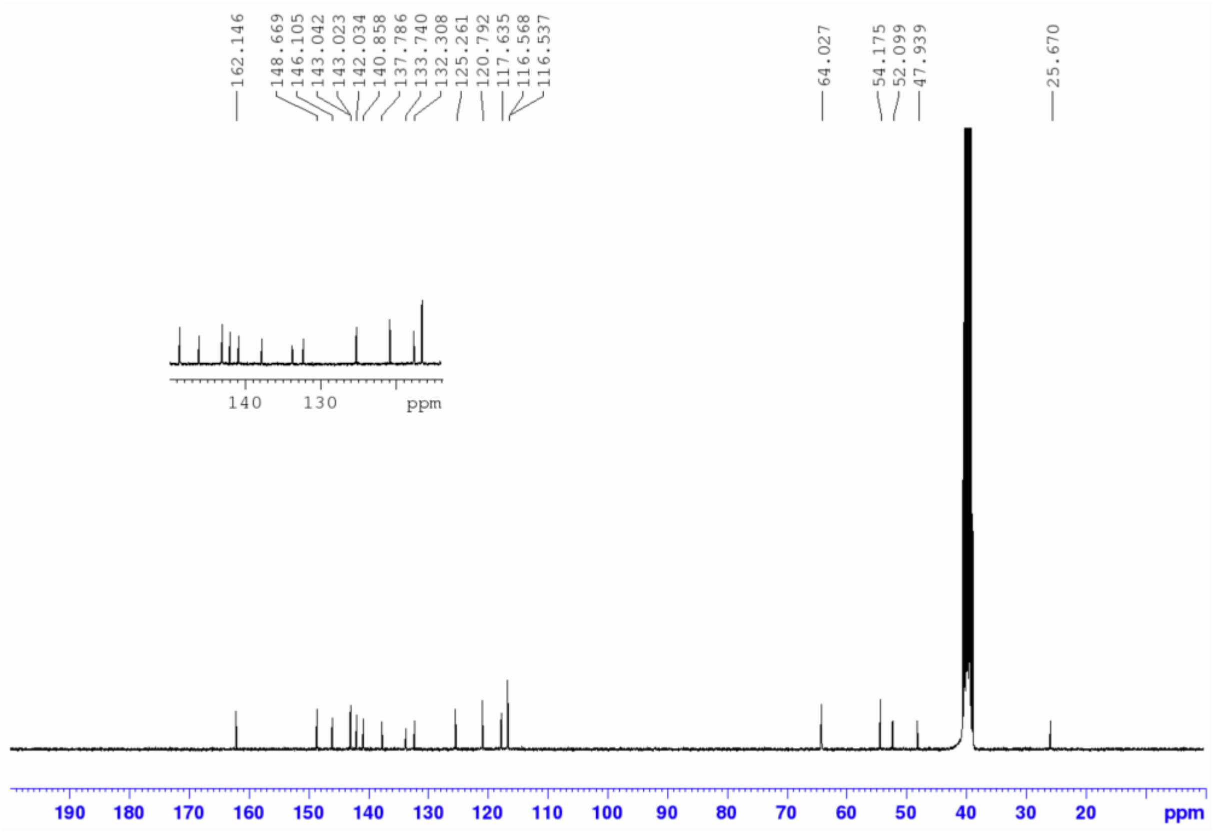

162.146
148.669
146.105
143.042
143.023
142.034
140.858
137.786
133.740
132.308
125.261
120.792
117.635
116.568
116.537
64.027
54.175
52.099
47.939
25.670
140
130
ppm
190
180
170
160
150
140
130
120
110
100
90
80
70
60
50
40
30
20
ppm

BDM71367 (**11a**)

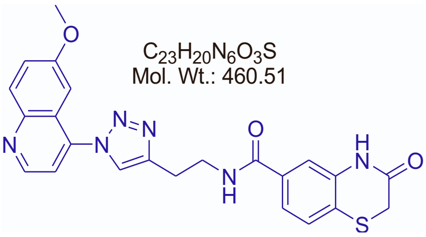

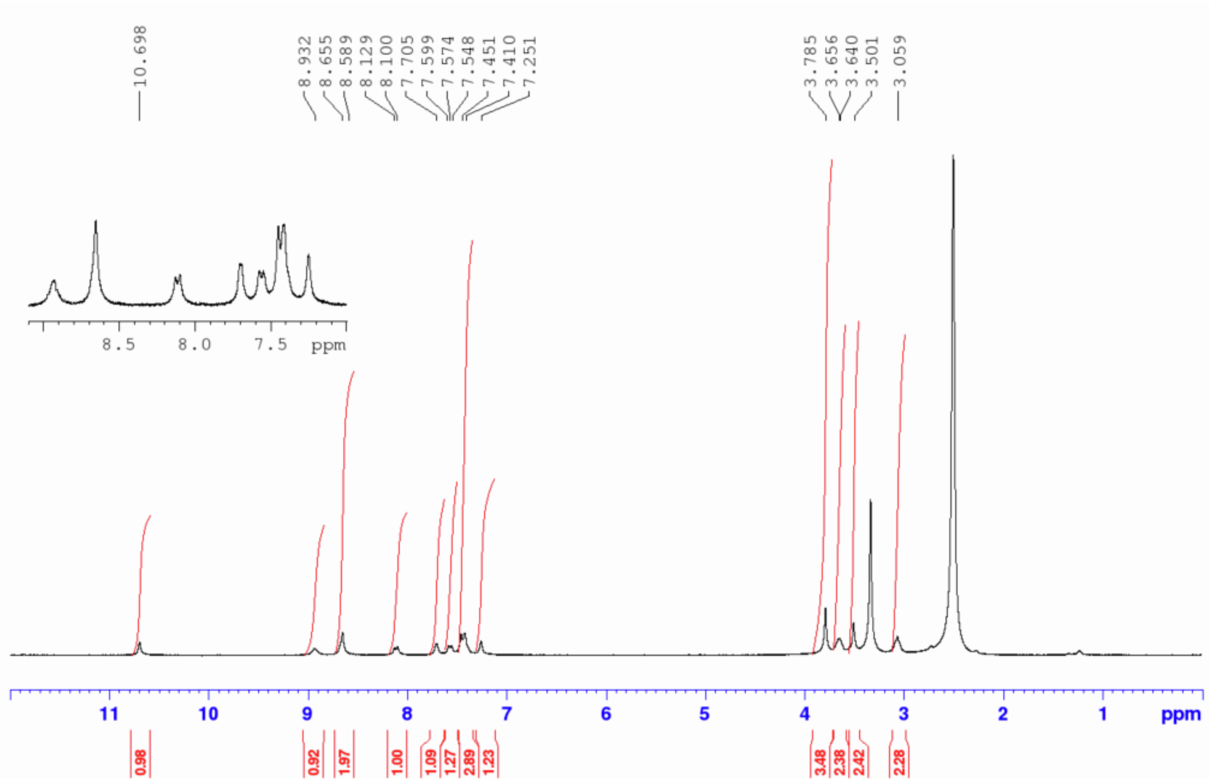

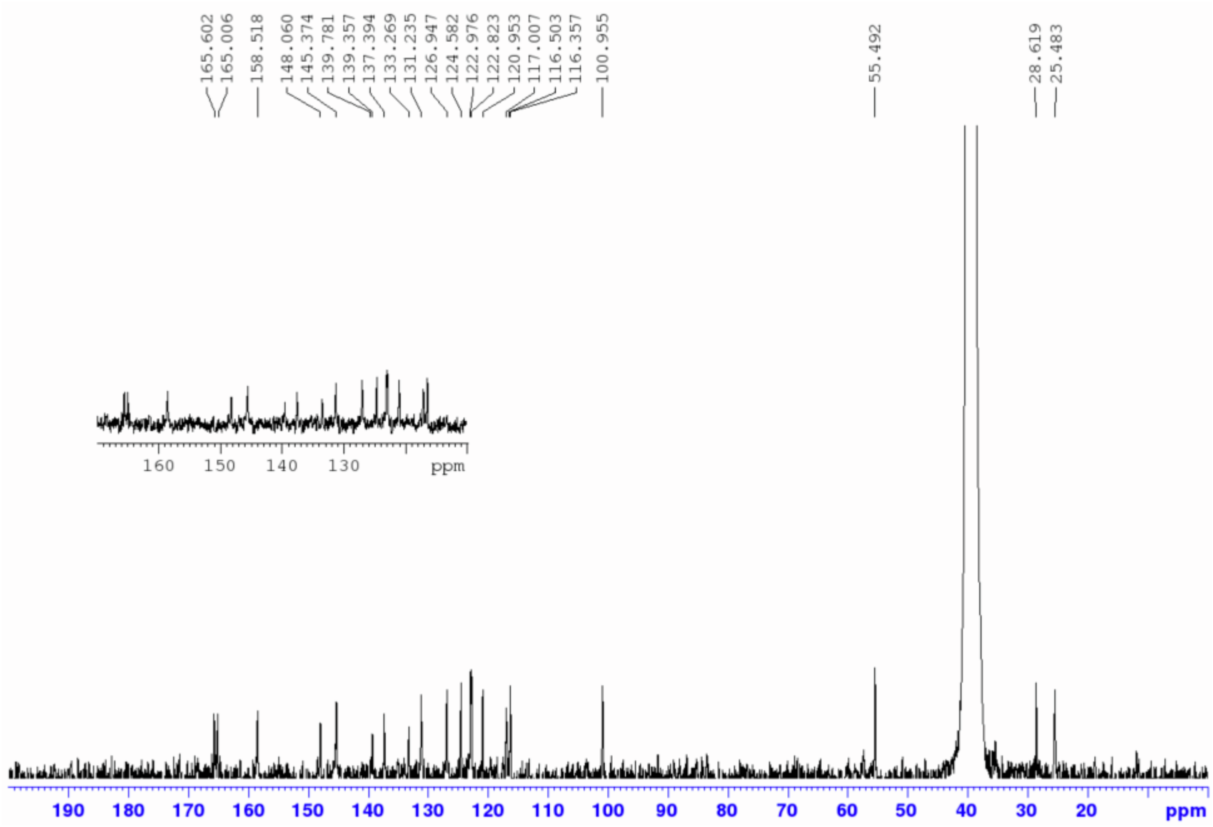

165.602
165.006
158.518
148.060
145.374
139.781
139.357
137.394
133.269
131.235
126.947
124.582
122.976
122.823
120.953
117.007
116.503
116.357
100.955
55.492
28.619
25.483
160 150 140 130 ppm
190 180 170 160 150 140 130 120 110 100 90 80 70 60 50 40 30 20 ppm

BDM71373 (**11b**)

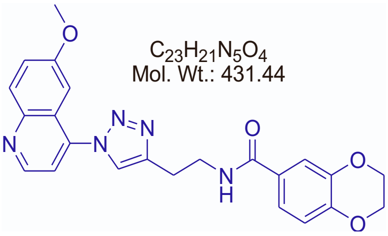

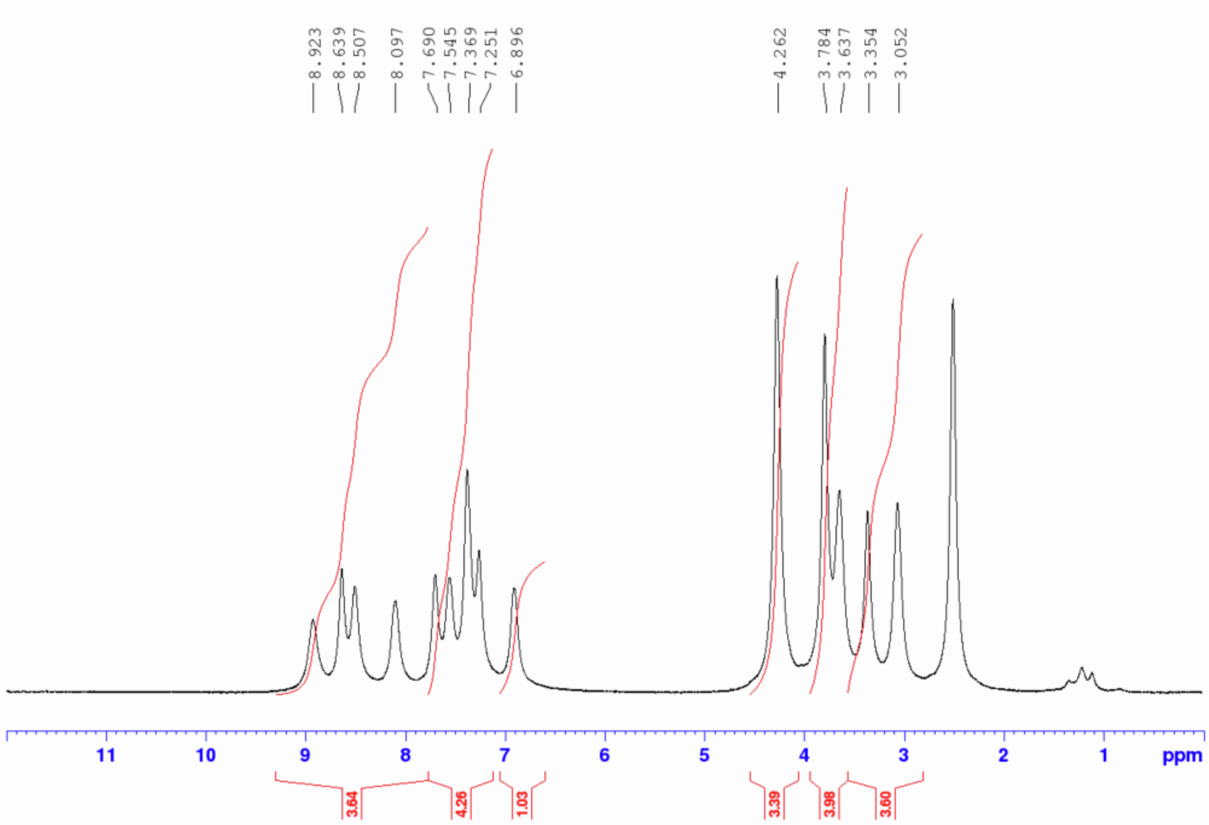

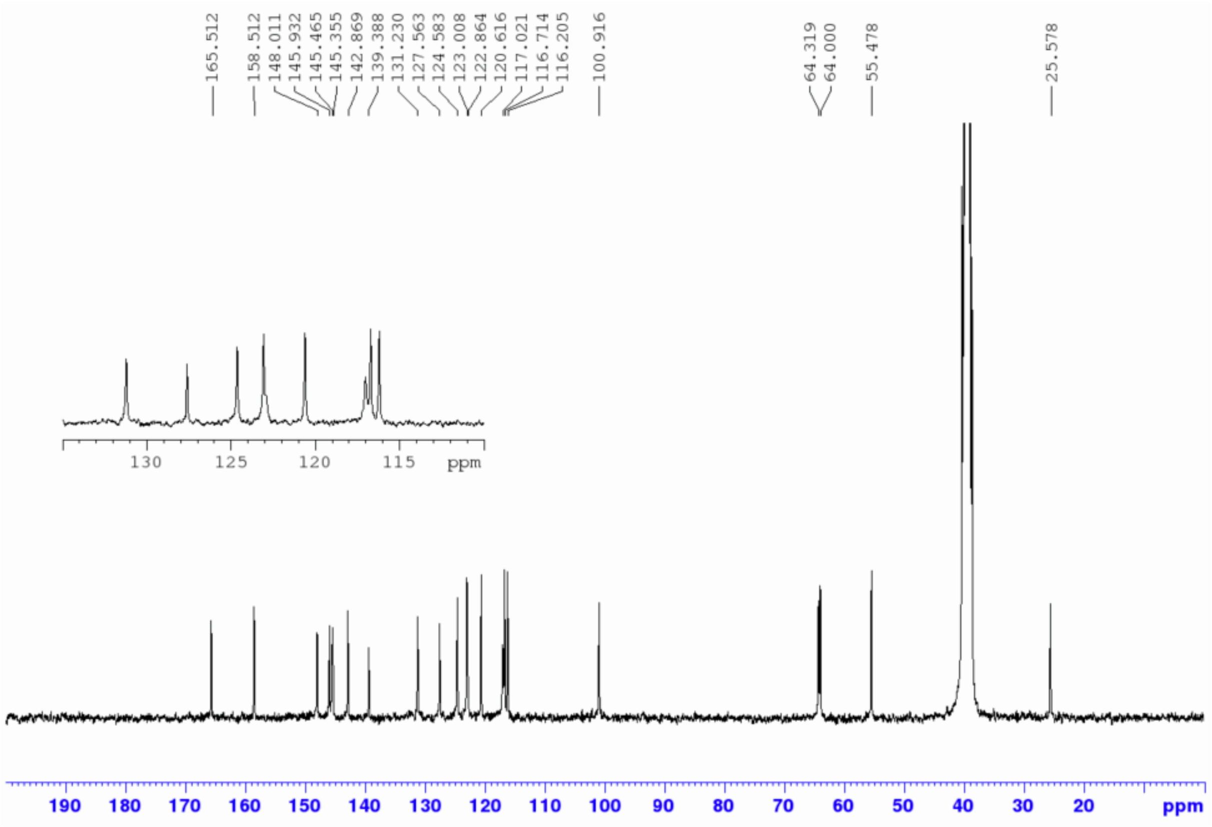
165.512
158.512
148.011
145.932
145.465
145.355
142.869
139.388
131.230
127.563
124.583
123.008
122.864
120.616
117.021
116.714
116.205
100.916
64.319
64.000
55.478
25.578
130
125
120
115
ppm
190
180
170
160
150
140
130
120
110
100
90
80
70
60
50
40
30
20
ppm

Supplementary references

(1) Larkin, M. A.; Blackshields, G.; Brown, N. P.; Chenna, R.; McGettigan, P. A.; McWilliam, H.; Valentin, F.; Wallace, I. M.; Wilm, A.; Lopez, R.; Thompson, J. D.; Gibson, T. J.; Higgins, D. G. Clustal W and Clustal X Version 2.0. *Bioinformatics* **2007**, *23* (21), 2947–2948. https://doi.org/10.1093/bioinformatics/btm404.

(2) Robert, X.; Gouet, P. Deciphering Key Features in Protein Structures with the New ENDscript Server. *Nucleic Acids Research* **2014**, *42* (W1), W320–W324. https://doi.org/10.1093/nar/gku316.
